# *S. aureus* exposure during cutaneous antigen sensitization causes basophil- and interleukin-4-dependent exaggerated food anaphylaxis

**DOI:** 10.1016/j.immuni.2025.09.001

**Published:** 2025-09-25

**Authors:** Mrinmoy Das, Mohammed Alasharee, Brian Woods, Saikat Mukherjee, Shira Kim, Megan Elkins, Jacqueline Ngo, Logan Magin, Maheshwor Timilshina, Juan Manuel Leyva-Castillo, Kenneth M. Murphy, Robert M. Anthony, Ana Flávia Santarine Laureano, George F. Murphy, Shannon McNamee, Frank Brombacher, Simon P. Hogan, Jerrold R. Turner, Shabnam Abtahi, Wanda Phipatanakul, Donald Y.M. Leung, Elena Goleva, Hans C. Oettgen, Mei Li, Janet Chou, Patrick M. Schlievert, Fred D. Finkelman, Raif S. Geha

**Affiliations:** 1 Division of Immunology, Boston Children’s Hospital, Department of Pediatrics, Harvard Medical School, Boston, MA, USA; 2 Division of Medical Research, SRM Medical College Hospital and Research Centre, SRM Institute of Science and Technology, Kattankulathur, India; 3 Department of Pathology and Immunology, Washington University School of Medicine, St. Louis, MO, USA; 4 Department of Medicine, Massachusetts General Hospital, Harvard Medical School, Boston, MA, USA; 5 Department of Surgery, Massachusetts General Hospital, Harvard Medical School, Boston, MA, USA; 6 Department of Pathology, Brigham and Women’s Hospital, Harvard Medical School, Boston, MA, USA; 7 University of Cape Town, Cape Town, South Africa; 8 Department of Pathology University of Michigan, Ann Arbor, MI, USA; 9 Department of Pathology, Brigham and Women’s Hospital and Harvard Medical School, Boston, MA, USA; 10 Department of Pediatrics, National Jewish Health, Denver, CO, USA; 11 Centre National de la Recherche Scientifique, Université de Strasbourg, Illkirch, France; 12 Department of Microbiology and Immunology, University of Iowa, Iowa City, IA, USA; 13 Division of Immunobiology, Department of Pediatrics, Division of Immunology, Department of Internal Medicine, University of Cincinnati College of Medicine, Cincinnati, OH, USA; 14 Present address: Vividion Therapeutic, Inc., San Diego, CA, USA; 15 Lead contact

## Abstract

The mechanism of the association of *S. aureus* skin colonization with food allergy in atopic dermatitis (AD) is unknown. Interleukin-4 (IL-4) plays an important role in food allergy. We found elevated serum IL-4 concentrations in AD patients with *S. aureus* skin colonization and food allergy. Using an AD mouse model, we demonstrated that epicutaneous application of antigen together with superantigen-producing *S. aureus*, or staphylococcal enterotoxin B (SEB), caused a heightened systemic antigen-specific T helper-2 (Th2) response and elevated serum IL-4 concentrations. T cell-derived IL-4 acted on intestinal epithelial cells to enhance intestinal permeability and anaphylaxis to enteral antigen challenge. CD40-dependent SEB binding to keratinocytes triggered IL-33 release, which caused T cells to produce IL-3 that elicited a basophil influx in skin-draining lymph nodes (dLNs). Basophil-derived IL-4 augmented Th2 cell polarization by antigen-bearing dendritic cells from skin dLNs. These results suggest therapeutic interventions that might attenuate food allergy in AD patients.

## In brief

*S. aureus* skin colonization is associated with food allergy in atopic dermatitis, but the mechanism of this association is unknown. Das et al. reveal that cutaneous exposure to *S. aureus* causes a basophil influx into skin-draining lymph nodes that drives a heightened T helper-2 response to simultaneously encountered antigen with increased serum IL-4 concentration, which enhances intestinal permeability, exaggerating oral anaphylaxis.

## Graphical Abstract

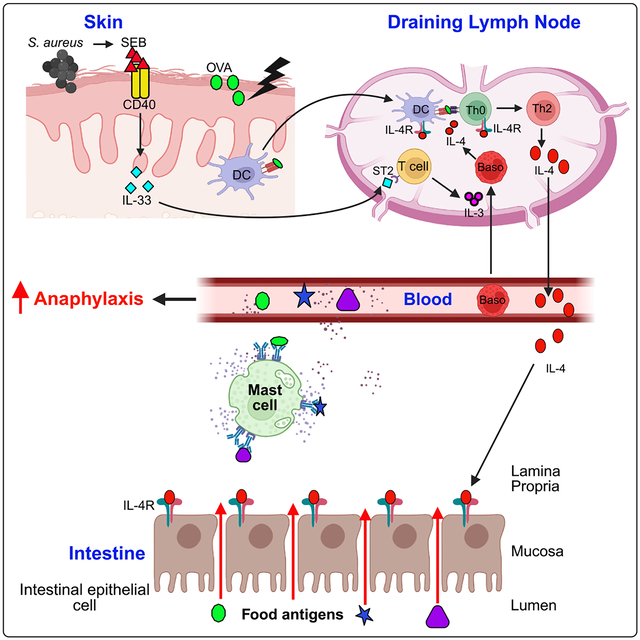

## INTRODUCTION

Food allergy affects 6%–8% of children and 3% of adults in the US.^1,2^ It manifests as immediate reactions to food ingestion that include urticaria, angioedema, and potentially fatal anaphylaxis, mediated by the release of mast cell (MC) mediators following recognition of food allergens by immunoglobulin E (IgE) antibodies bound to MC high-affinity IgE receptors.^3,4^ Sensitization to food allergens is not sufficient to drive food allergy. Not all individuals with food-specific IgE antibodies have food allergy.^5^ Factors other than IgE that dictate the severity of food allergy include intestinal MC numbers, interleukin-4 (IL-4), and intestinal permeability.^6–14^ MC mediator release in the gut increases intestinal permeability and thereby promotes antigen absorption into the circulation, which leads to increased systemic MC activation that causes anaphylaxis.^4,15^ IL-4 drives IgE production, promotes MC survival, increases the permeability of intestinal epithelial cells, and blocks the ability of T regulatory cells (Tregs) to suppress food allergy.^16–19^ Furthermore, IL-4 exacerbates anaphylaxis in mice challenged intravenously (i.v.) with anti-IgE monoclonal antibody (mAb).^6^

Skin is an important portal for sensitization to food.^20^ Food allergy is common in patients with atopic dermatitis (AD), a disease characterized by a disrupted skin barrier^21,22^ and associated with increased intestinal MC load and intestinal permeability.^23,24^ In mice, epicutaneous sensitization by application of antigen to skin that has been tape stripped to disrupt its barrier function results in IgE sensitization and allergic skin inflammation with features of AD, increased intestinal MC load and intestinal permeability, and IgE-mediated anaphylaxis following oral antigen challenge.^24–26^

The skin of AD patients is often colonized with *S. aureus* strains that produce superantigens, most commonly staphylococcal enterotoxin B (SEB).^27–29^ Skin colonization with *S. aureus* aggravates AD^30–36^ and is associated with higher concentrations of IgE antibodies to food antigens.^37–40^ There is a positive association between *S. aureus* skin colonization and food allergy in AD, independent of disease severity.^37,41^ Whether this is simply due to higher concentrations of IgE antibodies to food antigens and/or to non-antigen-specific enhancement of food allergy by *S. aureus* products is unknown.

Here, we show that serum IL-4 concentrations are elevated in patients with AD who have *S. aureus* skin colonization and food allergies. We demonstrate that application of ovalbumin (OVA) together with *S. aureus* or SEB to tape-stripped mouse skin resulted in exaggerated anaphylaxis following oral OVA challenge, a heightened systemic antigen-specific T helper-2 (Th2) cell response, and elevated serum IL-4 concentrations compared with application of OVA alone. Passive anaphylaxis experiments indicated that factors other than IgE contributed to selective exaggeration of oral anaphylaxis in mice sensitized to OVA with SEB. This was mediated by T cell-derived IL-4 acting on intestinal epithelial cells. SEB bound to keratinocytes in a CD40-dependent manner and triggered IL-33 release, which, in turn, caused T cells to produce IL-3 that subsequently elicited a basophil influx in skin-draining lymph nodes (dLNs). Basophil-derived IL-4 augmented Th2 cell polarization by dendritic cells (DCs) in dLNs that had captured cutaneously applied antigen.

## RESULTS

### Serum IL-4 concentrations are elevated in AD patients with *S. aureus* skin colonization and correlate with food allergy

**Figure 1.**
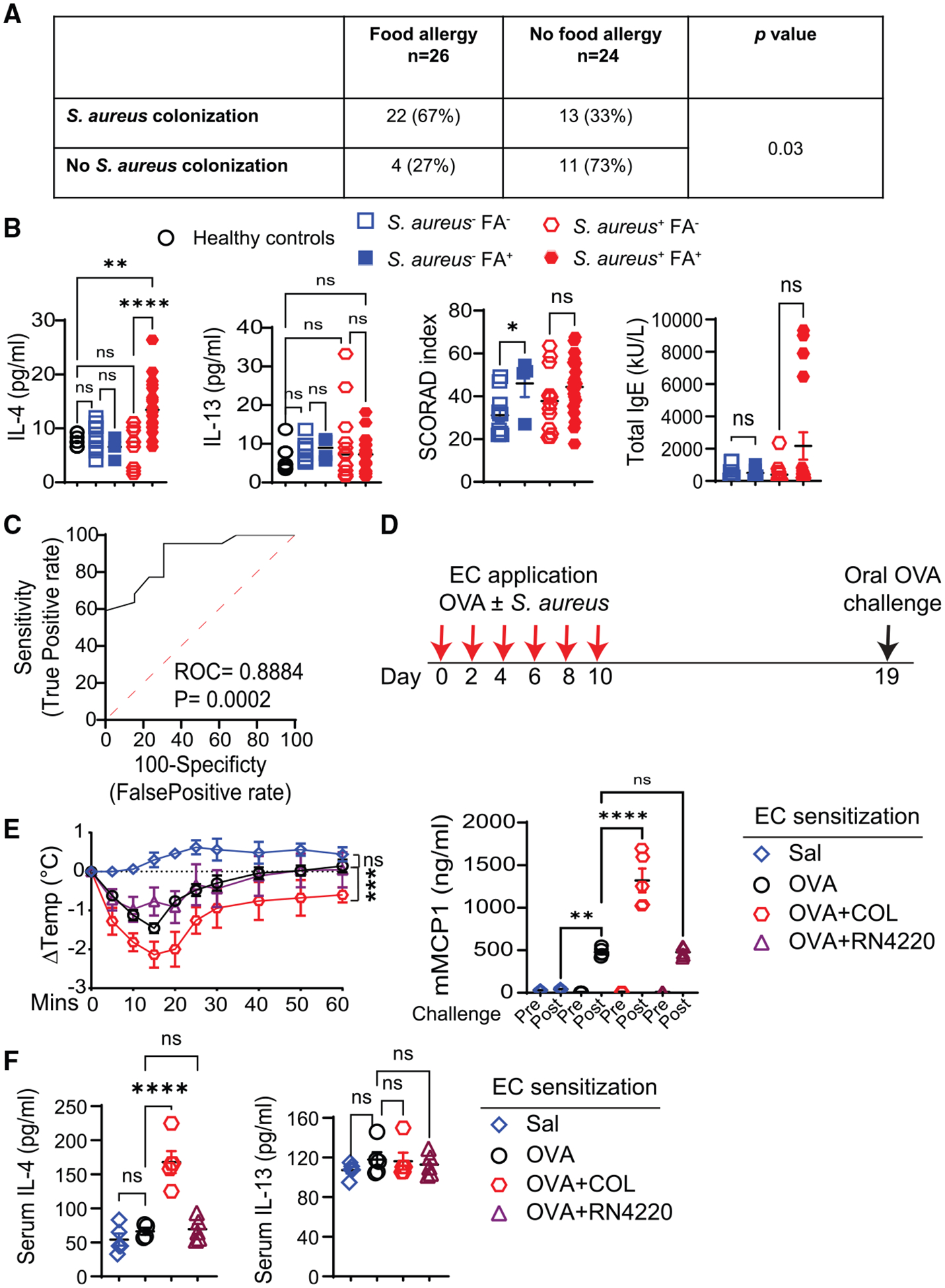
*S. aureus* skin colonization, food allergy, and serum IL-4 concentrations in AD patients and in an AD mouse model (A) Food allergy versus *S. aureus* skin colonization. (B) Serum concentrations of IL-4, IL-13, and IgE, and SCORAD in AD patients with or without *S. aureus* skin colonization and with or without food allergy. (C) ROC analysis of the relationship between serum IL-4 concentrations and food allergy in AD patients with *S. aureus* skin colonization. (D) Experimental protocol. (E and F) Change in body temperature post-OVA challenge (left) and serum mMCP-1 concentrations pre-challenge and 60 min post-challenge (right) (E), and serum IL-4 and IL-13 concentrations in epicutaneously sensitized mice (F), 4–5 mice/group. Data are presented as mean ± SEM. *p < 0.05, **p < 0.01, ***p < 0.001, ****p < 0.0001; ns, not significant by Fisher’s exact test in (A), one-way ANOVA with Tukey’s post hoc analysis in (B), (E) (right), (F), and repeated-measures one-way ANOVA (the Geisser-Greenhouse correction) in (E) (left). See also Figure S1.

IL-4 plays an important role in AD and food allergy. We examined the relationship between *S. aureus* skin colonization, serum IL-4 concentration, and food allergy in a cohort of 50 patients with AD. The characteristics of the patients are shown in Table S1. Thirty-five (70%) patients had *S. aureus* cultured from skin swabs (Figure 1A), consistent with published studies.^42^ Skin colonization with *S. aureus* significantly correlated with food allergy (odds ratio: 4.64, 95% confidence interval [CI]: 1.315–15.01, *p* < 0.03). Importantly, serum IL-4, but not IL-13, concentrations were significantly higher in patients with food allergy and *S. aureus* colonization than in those with *S. aureus* colonization but no food allergy (Figure 1B). Scoring AD (SCORAD) and IgE serum concentrations were comparable in the two groups. Food allergy was not associated with higher serum IL-4 concentrations in AD patients without detectable skin *S. aureus* colonization (Figure 1B). Receiver operated characteristic (ROC) analysis revealed that among patients with *S. aureus* skin colonization, an IL-4 concentration of >10.6 pg/mL had a specificity of 84.6% (95% CI: 57.8%–97.3%) and sensitivity of 68.2% (47.3%–83.6%) for food allergy (Figure 1C).

### Epicutaneous sensitization to antigen in the presence of *S. aureus* or SEB results in exaggerated oral anaphylaxis and elevated serum IL-4 concentrations

Tape stripping disrupts the skin barrier in mice, causing increased transepidermal water loss (Figure S1A) and allowing sensitization to antigen. We examined whether application of *S. aureus* together with OVA to tape-stripped skin of BALB/c mice increases serum IL-4 concentration and enhances anaphylaxis following oral OVA challenge. Figure 1D illustrates the experimental protocol used. As previously reported,^25,26^ mice sensitized with OVA underwent anaphylaxis following oral OVA challenge, evidenced by a significant drop in core body temperature and rise in serum mucosal MC protease-1 (mMCP-1) concentrations (Figure 1E). Sensitization with OVA in the presence of the COL strain, which produces multiple superantigens, but not of the mutagenized RN4220 strain, which produces no superantigens,^43–45^ resulted in exaggerated anaphylaxis following oral OVA challenge, as well as in increased serum concentrations of IL-4, but not IL-13, compared with sensitization with OVA alone (Figures 1E and 1F). *S. aureus* skin colonization was comparable between the COL and RN4220 strains (Figure S1B). Consistent with the role of superantigens in aggravating allergic skin inflammation,^46^ the latter was more severe in mice sensitized with the OVA + *S. aureus* COL strain compared with the OVA + *S. aureus* RN4220 strain, by histology and flow cytometry analysis of infiltrating cells (Figures S1C and S1D).

**Figure 2.**
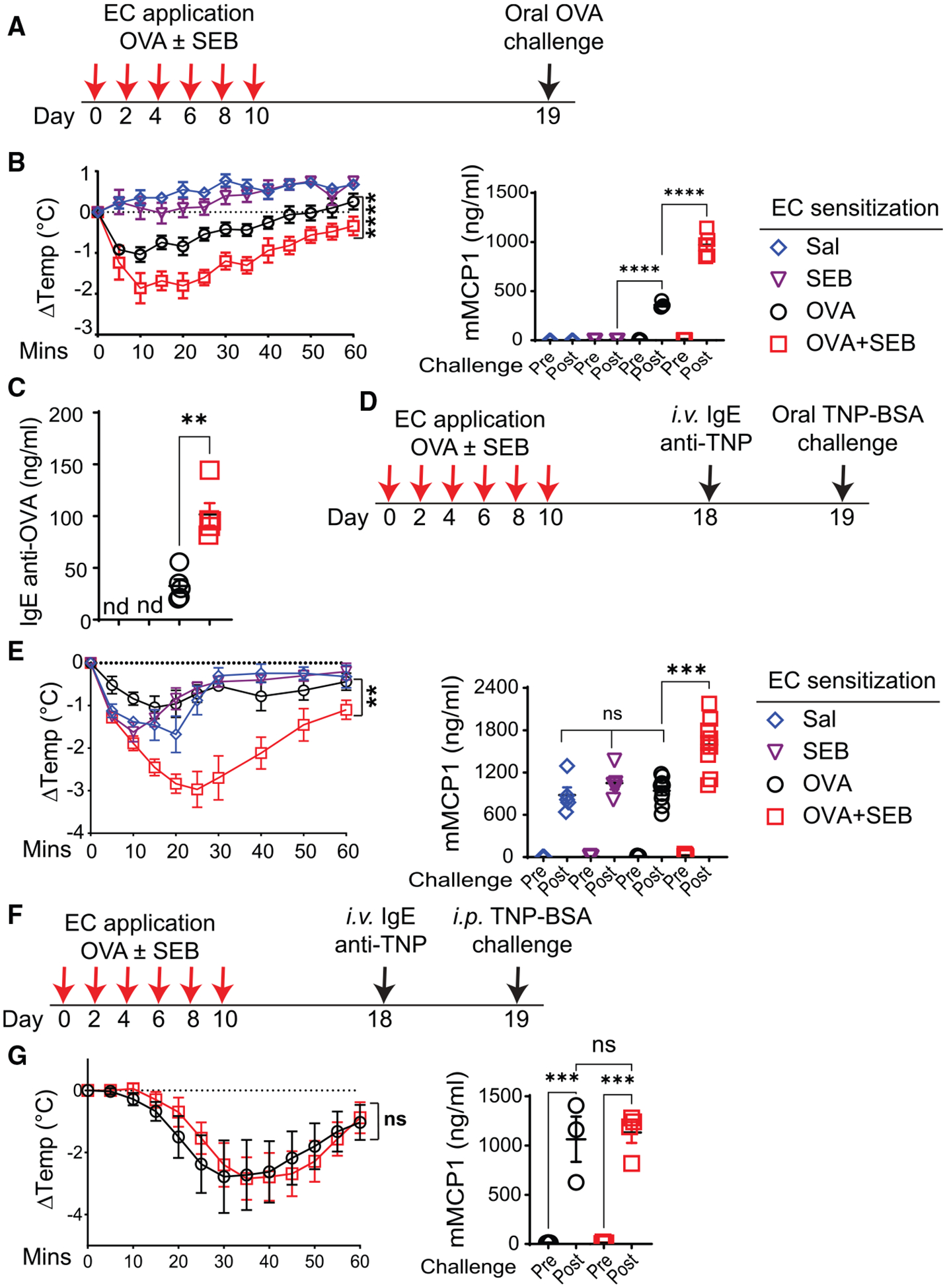
Epicutaneous sensitization to OVA in the presence of SEB results in exaggerated food anaphylaxis (A) Experimental protocol. (B) Change in body temperature post-OVA challenge (left) and serum mMCP-1 concentrations pre-challenge and 60 min post-challenge (right) with OVA in epicutaneously sensitized female BALB/c mice, 4–5 mice/group. (C) Serum IgE anti-OVA concentrations. (D and F) Experimental protocols for passive oral anaphylaxis (D) and passive systemic anaphylaxis (F). (E and G) Change in body temperature post TNP-BSA challenge (left) and serum mMCP-1 concentrations pre-challenge and 60 min post-challenge (right) in epicutaneously sensitized mice challenged orally (E) or i.p. (G) with TNP-BSA, 4–6 mice/group. A representative experiment out of 2 is shown for (C), (E), and (F), and a pool of 2 experiments is shown for (B). Data are presented as mean ± SEM. *p < 0.05, **p < 0.01, ***p < 0.001, ****p < 0.0001; ns, not significant by repeated-measures one-way ANOVA (the Geisser-Greenhouse correction) in (B) and (E) (left); (C) by Mann-Whitney t test; and (G) (left) by paired t test or one-way ANOVA with Tukey’s post hoc analysis in (B) (right), (E), (G) (right), and (D); nd, not detectable. See also Figure S2.

SEB is the superantigen most commonly produced by *S. aureus* strains, including COL, that colonize the skin of patients with AD.^27^ Epicutaneous sensitization of female BALB/c mice with OVA in the presence of purified SEB, hereafter referred to as OVA + SEB, resulted in exaggerated anaphylaxis following oral OVA challenge compared with epicutaneous sensitization with OVA (Figures 2A and 2B). Similar results were obtained using peanut antigen (Figure S2A). Exaggeration of anaphylaxis in OVA + SEB-sensitized mice was observed in male BALB/c and female C57BL/6J mice (Figures S2B and S2C). Oral anaphylaxis in OVA + SEB-sensitized mice, as in OVA-sensitized mice,^25^ was abolished in *Igh7*^−/−^ mice, which lack IgE (Figure S2D).

Mice sensitized with OVA on back skin and simultaneously exposed to SEB on tape-stripped ear skin, and mice exposed to SEB on tape-stripped back skin then sensitized 14 days later at the same site with OVA did not exhibit exaggerated anaphylaxis following oral OVA challenge (Figures S2E and S2F). Thus, SEB and antigen need to be applied together at the same skin site to cause exaggerated anaphylaxis following oral antigen challenge.

### Epicutaneous sensitization to OVA in the presence of *S. aureus* or SEB exacerbates passive oral anaphylaxis to an unrelated antigen

Mice epicutaneously sensitized with OVA + SEB, or with OVA + *S. aureus* COL but not OVA + *S. aureus* RN4220, had significantly higher OVA-specific IgE antibody concentrations compared with mice epicutaneously sensitized with OVA alone (Figures 2C and S2G). To determine whether factors other than IgE antibody contribute to the exaggerated oral anaphylaxis in these mice, we examined passive oral anaphylaxis. Following epicutaneous sensitization, mice received monoclonal IgE anti-trinitrophenyl (TNP) antibody i.v. and then were orally challenged 24 h later with TNP-BSA (Figure 2D). Mice sensitized with OVA, SEB, or saline demonstrated comparable passive oral anaphylaxis (Figures 2F and 2G). Mice sensitized with OVA + SEB or with OVA + *S. aureus* COL, but not OVA + *S. aureus* RN4220, demonstrated exaggerated passive oral anaphylaxis compared with mice sensitized with OVA alone (Figures 2E, S2H, and S2I), indicating that factors beyond differences in antigen-specific IgE antibody (Ab) concentrations or affinity contributed to the increased susceptibility to oral anaphylaxis. We subsequently used passive oral anaphylaxis to dissect these factors.

### Epicutaneous sensitization to OVA + SEB does not exaggerate passive anaphylaxis to i.p. or i.d. antigen challenge

We investigated whether the increased susceptibility to anaphylaxis of mice epicutaneously sensitized to OVA + SEB was generalized or specific to oral challenge. We first examined passive systemic anaphylaxis. Following epicutaneous sensitization, mice were passively sensitized with IgE anti-TNP and then challenged 24 h later by intraperitoneal (i.p.) injection of TNP-BSA (Figure 2F). PSA was comparable in the two groups (Figure 2G). We also examined passive cutaneous anaphylaxis. The right ears of epicutaneously sensitized mice were passively sensitized by intradermal (i.d.) injection of IgE anti-TNP, and the left ears were injected i.d. with PBS as a control. 24 h later, TNP-BSA together with Evans blue dye was administered i.v., and 30 min later, the Evans blue content of ears was determined (Figure S2J). Evans blue content was significantly higher in IgE anti-TNP injected ears compared with PBS injected ears but was comparable in anti-TNP injected ears of mice sensitized with OVA + SEB versus OVA alone (Figure S2K). These results indicate that epicutaneous sensitization to OVA + SEB selectively exaggerates anaphylaxis in response to oral antigen challenge.

### Epicutaneous sensitization to OVA + SEB promotes the antigen-specific systemic Th2 cell response

**Figure 3.**
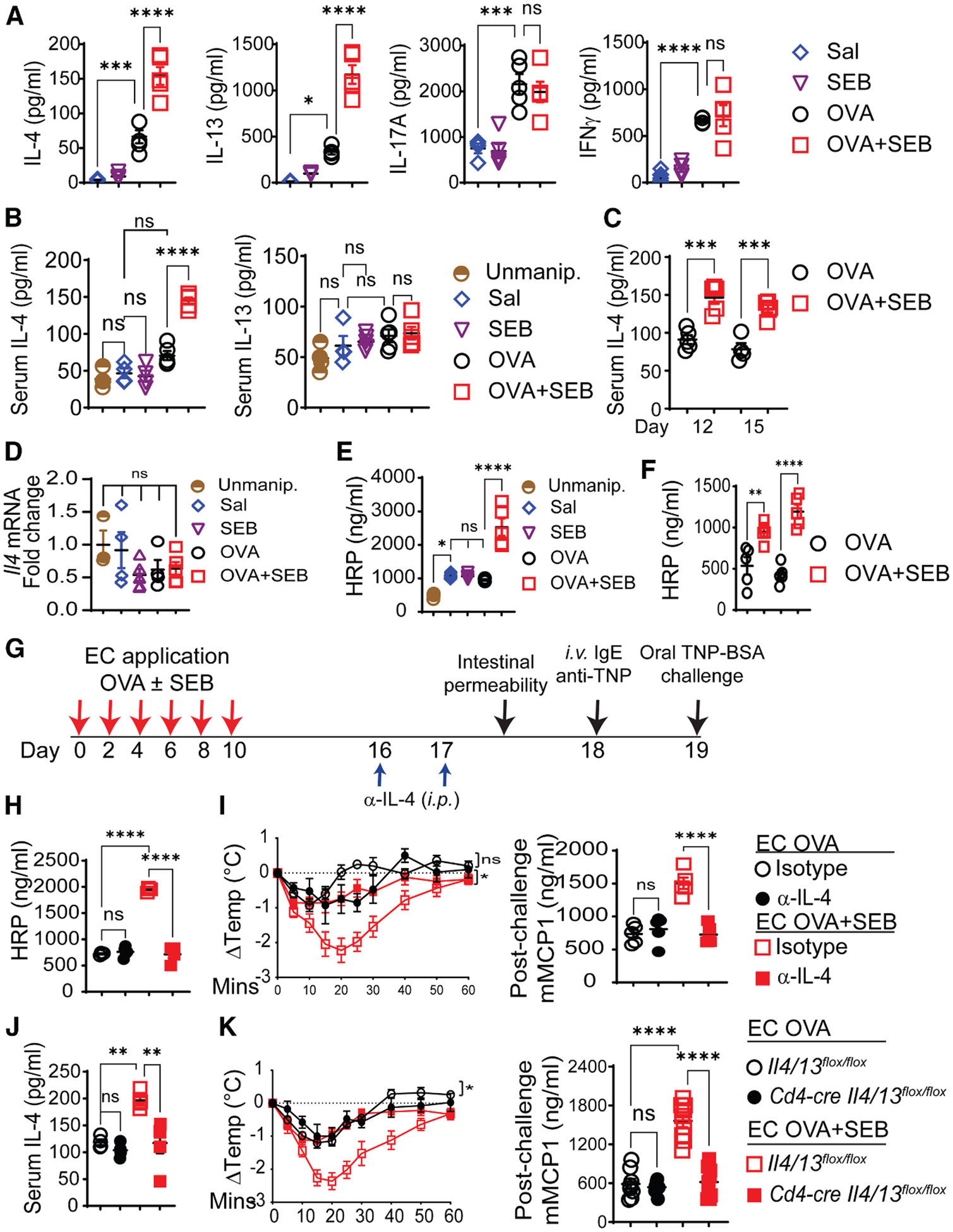
Epicutaneous sensitization to OVA + SEB enhances the systemic Th2 response to antigen, elevates serum IL-4 concentrations, and drives IL-4-dependent increases in intestinal permeability and oral anaphylaxis (A) Cytokine secretion by OVA-stimulated splenocytes from epicutaneously sensitized mice, 4–5 mice/group. (B) Serum IL-4 and IL-13 concentrations on day 12 of epicutaneous sensitization and unmanipulated (Unmanip.) controls, 4–6 mice/group. (C) Serum IL-4 concentrations on days 15 and 19 of epicutaneous sensitization. (D) Intestinal *II4* mRNA expression on day 19 of epicutaneous sensitization. (E) Serum HRP concentrations 20 min after oral administration of HRP day 19 of epicutaneous sensitization in 4–5 mice/group. (F) Serum HRP concentrations on days 12 and 15 of epicutaneous sensitization. (G) Experimental protocol for epicutaneous sensitization, anti-IL-4 treatment, intestinal permeability measurement, and passive oral anaphylaxis. (H and I) Serum HRP concentrations (H), change in body temperature (middle), and serum concentrations of mMCP-1 (right) post-oral challenge (I), 5 mice/group. (J and K) Serum IL-4 concentrations (J), passive oral anaphylaxis with post-challenge change in body temperature (K, left), and serum mMCP-1 concentrations (K, right) in epicutaneously sensitized *Cd4-creIl4/13*^*flox/flox*^ mice and *Il4/13*^*flox/flox*^ controls, 4–5 mice/group. A representative experiment out of 2 is shown for (A)–(J), and a pool of 2 experiments is shown for (K). Data are presented as mean ± SEM. *p < 0.05, **p < 0.01, ***p < 0.001, ****p < 0.0001 by one-way ANOVA with Tukey’s post hoc analysis in (A)–(E), (H), (I) (right), (J), and (K) (right) and repeated-measures one-way ANOVA (the Geisser-Greenhouse correction) in (I) (left) and (K) (left); ns, not significant. See also Figure S3.

Splenocytes from mice epicutaneously sensitized with OVA secrete more IL-4, IL-13, IL-17A, and interferon (IFN)γ in response to *in vitro* stimulation with OVA compared with mice epicutaneously sensitized with saline.^47,48^ Secretion of IL-4 and IL-13, but not IL-17A or IFNγ, by OVA-stimulated splenocytes was significantly higher in mice sensitized with OVA + SEB compared with OVA alone (Figure 3A). Importantly, mice epicutaneously sensitized with OVA + SEB, but not OVA alone, SEB, or saline, demonstrated on day 12 a significant increase in serum concentrations of IL-4, but not IL-13, compared with unmanipulated mice (Figure 3B). Serum IL-4 concentrations were also significantly higher in OVA + SEB versus OVA-exposed mice on days 15 and 19 (Figure 3C). There was no increase in *Il4* mRNA expression in the intestine of OVA + SEB-sensitized mice (Figure 3D).

### IL-4 drives increased intestinal permeability and exaggerated oral anaphylaxis following epicutaneous sensitization with OVA + SEB

Intestinal permeability plays an important role in food allergy and is increased in patients with AD and with food allergy.^13,14,24^ Intestinal permeability was examined by measuring serum horse-radish peroxidase (HRP) concentrations 20 min after gavaging mice with HRP. Intestinal permeability immediately before oral challenge (day 19) was higher in OVA + SEB-exposed mice compared with mice exposed to OVA, SEB, or saline (Figure 3E). Intestinal permeability in the latter three groups exceeded intestinal permeability in unmanipulated mice, consistent with the reported increase in intestinal permeability following tape stripping.^24^ Intestinal permeability was also significantly higher in OVA + SEB- versus OVA-exposed mice on days 12 and 15 (Figure 3F).

IL-4 and IL-13 have been reported to promote intestinal permeability.^18,19,49,50^ To investigate their individual roles, blocking mAbs to mouse IL-4 or IL-13, or IgG1 isotype control, were administered i.p. on days 16 and 17, intestinal permeability was measured on day 18, followed by passive sensitization and measurement of passive oral anaphylaxis on day 19 (Figure 3G). IL-4, but not IL-13, blockade abolished the increased intestinal permeability and enhanced in OVA + SEB-sensitized mice (Figures 3H, 3I, S3A, and S3B). Neither blockade had a detectable effect on intestinal permeability or passive oral anaphylaxis in mice sensitized with OVA alone (Figures 3H, 3I, S3A, and S3B). IFN-γ or/and IL-17A blockade had no effect of intestinal permeability or passive oral anaphylaxis in either OVA + SEB- or OVA-sensitized mice (Figures S3C and S3D). These results indicate that IL-4 plays a critical role in the increased intestinal permeability and exaggerated oral anaphylaxis in mice epicutaneously sensitized to antigen in the presence of SEB.

### T cell-derived IL-4 drives the exaggerated passive oral anaphylaxis in mice epicutaneously sensitized with OVA in the presence of SEB

Since IL-4, but not IL-13, was responsible for the enhanced oral anaphylaxis in OVA + SEB-sensitized mice, we were able to use cre-mediated deletion in *Il4/13*^*flox/flox*^ mice to examine the relevant source of IL-4 that drives the exaggerated oral anaphylaxis in these mice. IL-4 derived from T cells, type 2 innate lymphoid cells (ILC2s), and MCs may contribute to oral anaphylaxis.^16,17,51^ Both the increase in IL-4 serum concentrations and enhanced passive oral anaphylaxis were abolished in OVA + SEB-sensitized *Cd4-cre Il4/13*^*flox/flox*^ mice, which selectively lack IL-4 and IL-13 in T cells, compared with *Il4/13*^*flox/flox*^ controls (Figures 3J and 3K). By contrast, serum IL-4 concentrations and passive oral anaphylaxis in OVA + SEB-sensitized *Rora-cre Il4/13*^*flox/flox*^ mice, which lack IL-4 and IL-13 in ILC2s, and *Mctp5-cre Il4/13*^*flox/flox*^ mice, which lack IL-4 and IL-13 in MCs, were comparable to *Il4/13*^*flox/flox*^ controls (Figures S3E–S3H). Deletion of IL-4 and IL-13 in T cells, ILC2s or MCs had no significant effects on serum IL-4 concentrations or passive oral anaphylaxis in mice sensitized with OVA alone (Figures 3J, 3K, and S3E–S3H). These results suggest that T cell-derived IL-4 drives the rise in serum IL-4 concentrations and the enhanced passive oral anaphylaxis in mice epicutaneously sensitized to antigen in the presence of SEB.

Higher percentages of splenic CD4^+^IL-4^+^ cells were detected on day 19 in OVA + SEB-sensitized compared with OVA-sensitized mice (Figure S3I). Depletion of CD4^+^ cells by administration of anti-CD4 mAb on days 15 and 17 abolished the increases in serum IL-4 concentrations, intestinal permeability, and passive oral anaphylaxis on day 19 in OVA + SEB-sensitized mice (Figures S3J–S3N). OVA + SEB-sensitized mice transgenic for a T cell receptor (TCR) that recognizes myelin oligodendrocyte glycoprotein (MOG) but not OVA (TCR^MOG^) did not demonstrate a rise in serum IL-4 or exaggerated passive oral anaphylaxis (Figures S3O and S3P). These findings suggest antigen-dependent persistent activation of Th2 cells in OVA + SEB-sensitized wild-type (WT) mice.

### IL-4 acts on intestinal epithelial cells to promote intestinal permeability and passive oral anaphylaxis following epicutaneous sensitization with OVA + SEB

**Figure 4.**
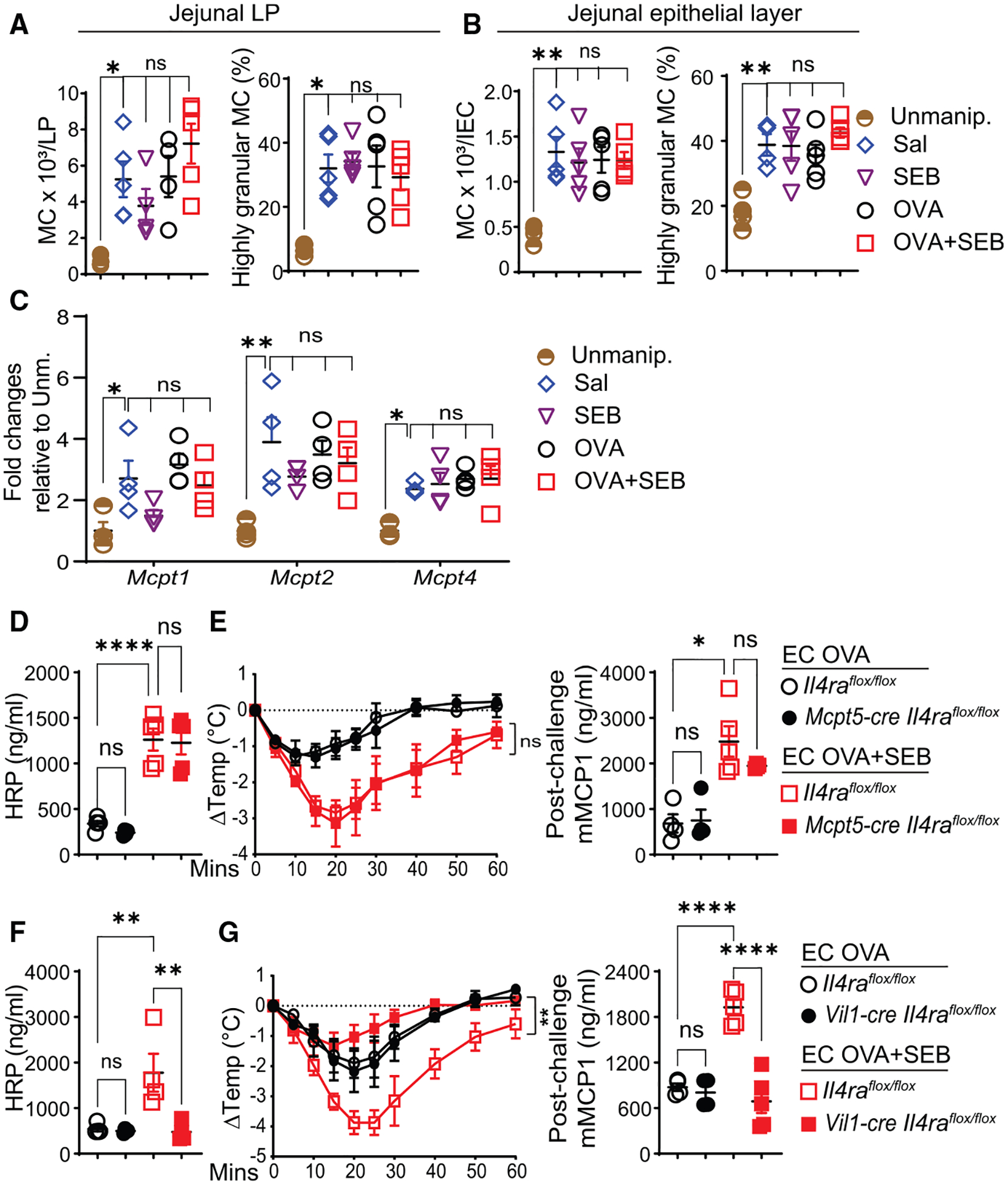
Enhanced food anaphylaxis and intestinal permeability in mice epicutaneously sensitized in the presence of SEB are dependent on IL-4Rα expression by intestinal epithelial cells (A and B) Number of CD45^+^Lin^−^c-kit^+^IgE^+^ MCs and percentage of side scatter (SSC)^high^ MCs in jejunal LP (A) and epithelial layer (B) of epicutaneously sensitized mice and Unmanip. controls, 4–5 mice/group. (C) mRNA expression of *Mcpt1*, *Mcpt2*, and *Mcpt4* in the jejunum of epicutaneously sensitized mice relative to Unmanip. controls. (D–H) Serum HRP concentrations following gavage with HRP pre-challenge (D and F) and post-challenge change in body temperature (E and G, left) and serum mMCP-1 concentrations (E and G, right) in *Mcpt5-creIl4ra*^*flox/flox*^ mice and *Il4ra*^*flox/flox*^ controls (D and E) and *Vil1-creIl4ra*^*flox/flox*^ mice and *Il4ra*^*flox/flox*^ controls (F and G) subjected to passive oral anaphylaxis, 4–5 mice/group. In all panels, a representative experiment out of 2 is shown. Data are presented as mean ± SEM. *p < 0.05, **p < 0.01, ***p < 0.001, ****p < 0.0001 by one-way ANOVA with Tukey’s post hoc analysis in (A)–(G) (right); repeated-measures one-way ANOVA (the Geisser-Greenhouse correction) in (E) and (G) (left); or one-way ANOVA with Dunn’s post hoc test in (F) right; ns, not significant. See also Figure S4.

IL-4 could potentially target intestinal epithelial cells, MCs, and vascular endothelial cells to exaggerate oral anaphylaxis in OVA + SEB-sensitized mice. Tape stripping triggers intestinal MC expansion associated with increased intestinal permeability and enhanced passive oral anaphylaxis.^24,25^ The number and granularity of CD45^+^Lin^−^c-Kit^+^IgE^+^ MCs in the jejunal lamina propria (LP) and epithelial layer, jejunal expression of genes encoding the MC proteases *Mcpt1* and *Mcpt2*, expressed primarily by LP MCs, and *Mcpt4*, expressed primarily by intraepithelial MCs,^52^ were comparably increased in mice exposed to OVA + SEB, OVA, SEB, or saline relative to unmanipulated mice (Figures 4A–4C).

Further, MC numbers in the duodenum and ileum were comparable in OVA + SEB- and OVA-sensitized mice (Figure S4A). Importantly, intestinal permeability and passive oral anaphylaxis were comparable in OVA + SEB-sensitized, *Mcpt5-creIl4ra*^*flox/flox*^ mice, which lack *Il4ra* expression in MCs, and *Il4ra*^*flox/flox*^ controls (Figures 3D, 3E, and S4B). Furthermore, passive oral anaphylaxis was comparable in OVA + SEB-sensitized *Cdh5-creIl4ra*^*flox/flox*^ mice, which selectively lack *Il4ra* in vascular endothelial cells, and *Il4ra*^*flox/flox*^ controls (Figure S4C).

In contrast to mice that lack *Il4ra* in MCs or vascular endothelial cells, OVA + SEB-sensitized *Vil1-creIl4ra*^*flox/flox*^ mice, which selectively lack interleukin-4 receptor alpha (IL-4Rα) in intestinal epithelial cells, did not demonstrate increased intestinal permeability and passive oral anaphylaxis compared *Il4ra*^*flox/flox*^ controls, although they demonstrated increased serum IL-4 concentrations (Figures 4F, 4G, and S4D). *Il4ra* deletion in intestinal epithelial cells had no effect on intestinal permeability, passive oral anaphylaxis, or serum IL-4 concentrations in OVA-sensitized mice (Figures 4F, 4G, and S4D).

Enterocytes are the major subtype of intestinal epithelial cells, and IL-4 increases the permeability of enterocytic cell monolayers,^53,54^ suggesting that IL-4 targets enterocytes to cause increased intestinal permeability and passive oral anaphylaxis in OVA + SEB-sensitized mice. Tuft cells, Paneth cells, and goblet cells also express IL-4 receptor (IL-4R). Like WT mice, OVA + SEB-sensitized *Pou2f3*^−/−^ mice, which lack tuft cells, demonstrated increased intestinal permeability and passive oral anaphylaxis compared with OVA-sensitized controls (Figures S4E and S4F), ruling out a major role for tuft cells. We cannot rule out a role for Paneth cells and goblet cells. Altogether, the above findings suggest that the previously described IL-33-, tuft cell-, IL-25-, ILC2-, and IL-4-dependent intestinal MC expansion triggered by tape stripping^24^ is operative in OVA + SEB-sensitized mice, and that the elevated IL-4 concentrations elicited through epicutaneous sensitization with OVA + SEB bypass tuft cells and directly signal intestinal epithelial cells to increase intestinal permeability and exaggerate anaphylaxis.

### DCs from dLNs of skin exposed to OVA + SEB promote Th2 polarization and demonstrate increased IL-4R signaling

**Figure 5.**
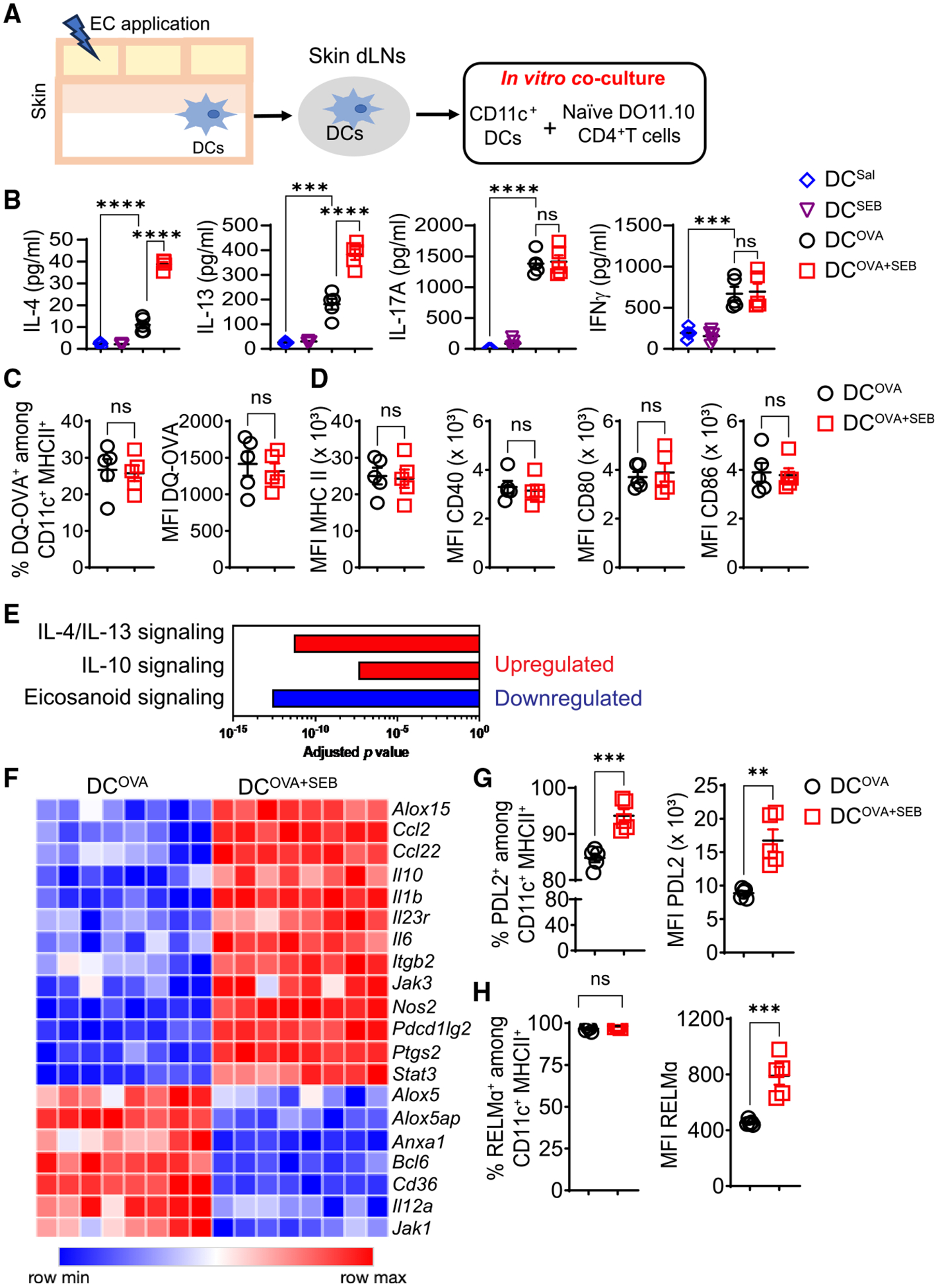
Cutaneous exposure to OVA + SEB enhances Th2 cell polarization by skin dLN DCs (A) Experimental protocol for testing Th2 cell polarization by CD11c^+^ cells from dLNs of mouse skin exposed to OVA + SEB (DC^OVA + SEB^), OVA alone (DC^OVA^), SEB alone (DC^SEB^), or saline (DC^Sal^). (B) Cytokine secretion, 4–5 mice/group. (C) Percentage of DQ-OVA^+^ CD11c^+^MHC class II^high^ skin in skin dLNs of mice exposed to DQ-OVA (DC^DQ-OVA^) or DQ-OVA + SEB (DC^DQ-OVA + SEB^) and mean fluorescence intensity (MFI) of DQ-OVA, 5 mice/group. (D) MFI of MHC class II, CD40, CD80, and CD86 in DC^DQ-OVA^ and DC^DQ-OVA + SEB^, 5 mice/group. (E and F) Ingenuity pathway analysis (E) and gene expression heatmap of IL-14/IL-13 regulated genes (F) in DC^OVA + SEB^ and DC^OVA^, 8 mice/group. (G and H) Percentage and MFI of PDL2^+^ cells (G) and RELMα^+^ cells (H) among CD11c^+^MHC class II^high^ DC^OVA + SEB^ and DC^OVA^, 5 mice/group. For (B)–(D), (G), and (H), a representative experiment out of 2 is shown. Data are presented as mean ± SEM. ***p < 0.001, ****p < 0.0001; ns, not significant by one-way ANOVA with Tukey’s post hoc analysis in (B) and Student’s t test in (C), (D), (G), and (H). See also Figure S5.

DCs that capture antigen in the skin migrate to dLNs where they drive the differentiation of antigen-specific naive T cells into Th cells. We investigated whether DCs from dLNs of skin exposed to OVA + SEB were more potent in driving Th2 cell differentiation compared with DCs from skin exposed to OVA alone. DCs isolated from dLNs 24 h after cutaneous application of OVA + SEB (DC^OVA + SEB^), OVA alone (DC^OVA^), SEB (DC^SEB^), or saline (DC^Sal^) were co-cultured with naive OVA-specific CD4^+^ T cells isolated from spleens of TCR-OVA transgenic D0.11.10 mice without addition of exogenous OVA (Figure 5A). DC^OVA + SEB^ caused secretion by T cells of significantly higher amounts of IL-4 and IL-13, but not IL-17A and IFN-γ, compared with DC^OVA^ (Figure 5B). DC^SEB^ did not cause T cells to secrete cytokines (Figure 5B); however, as expected, the addition of SEB caused DCs^SEB^ to drive cytokine secretion by T cells (Figure S5A). These results suggest that DCs in dLNs of SEB-exposed skin do not display SEB on their surface. This was verified by examining DCs in dLNs for surface biotin 24 h after application of biotinylated SEB to tape-stripped skin (Figure S5B). Thus, potentiation of Th2 cell polarization by DC^OVA + SEB^ was not due to engagement of Vβ8 on D0.11.10 CD4^+^ T cells by SEB.

DCs that have recently migrated from skin to dLNs express high amounts of major histocompatibility complex (MHC) class II molecules.^55^ DQ-OVA is a self-quenched OVA boron-dipyrro-methene (BODIPY)-fluorescent dye conjugate that exhibits bright green fluorescence upon proteolytic degradation in lysosomes. The numbers of CD11c^+^MHC class II^high^DQ-OVA^+^ DCs, their DQ-OVA fluorescence, and their surface expression of MHC class II, CD40, CD80, and CD86 were comparable in dLNs of mice cutaneously exposed to OVA-DQ + SEB versus OVA-DQ alone (Figures 5C and 5D).

To examine whether skin exposure to OVA + SEB alters the expression of genes that regulate the ability of DCs to drive Th2 cell polarization, we compared the gene expression profile of DC^OVA + SEB^ and DC^OVA^ isolated from skin dLNs. Among the pathways enriched for differentially expressed genes (fold change ≥ 2, false discovery rate [FDR] < 0.05), we defined significantly upregulated versus downregulated pathways as those with a *Z* score either ≥2 or ≤−2, respectively. The most upregulated pathways in DC^OVA + SEB^ were IL-4/IL-13 signaling and IL-10 signaling (Figure 5E). Consistent with increased IL-4/IL-13 signaling and enhanced Th2 cell polarizing capacity, DC^OVA + SEB^ showed increased expression of *Pdcd1lg2 Il10* and *Il6*,^56–62^ and decreased expression of *Il12a*.^63–65^ The most significantly downregulated pathway in DC^OVA + SEB^ was eicosanoid signaling. Among genes in this pathway, *Alox5*, as previously reported,^66^ as well as *Alox5AP* and *Anxa1*, were downregulated by IL-4/IL-13 signaling in DC^OVA + SEB^ (Figure 5F).

We also examined CD11c^+^MHC class II^high^ DCs from skin dLNs for expression of proteins known to be important for Th2 cell polarization and for stabilizing the interactions between DCs and CD4^+^ T cells. These include programmed cell death-ligand 2 (PDL2), encoded by *Pdcd1lg2*, resistin-like molecule alpha (RELMα), also upregulated by IL-4 and important for Th2 cell polarization,^67^ and integrin CD301b, which promotes formation of macroclusters between DCs and CD4^+^ T cells.^68^ The percentage of PDL2^+^ cells and PDL2 expression, but not the percentage of CD301b^+^ cells, or CD301b expression, were significantly increased in CD11c^+^MHC class II^high^ cells from dLNs of OVA + SEB-exposed skin compared with OVA-exposed skin (Figures 5G and S5C). Virtually all CD11c^+^MHC class II^high^ cells expressed intracellular RELMα; however, RELMα expression was significantly higher in CD11c^+^MHC class II^high^ cells from dLNs of OVA + SEB-exposed skin compared with OVA-exposed skin (Figure 5G). Collectively, these results indicate that the addition of SEB robustly increased IL-4R signaling in DCs.

### Cutaneous exposure to SEB causes accumulation of basophils in dLNs that potentiate Th2 cell polarization

**Figure 6.**
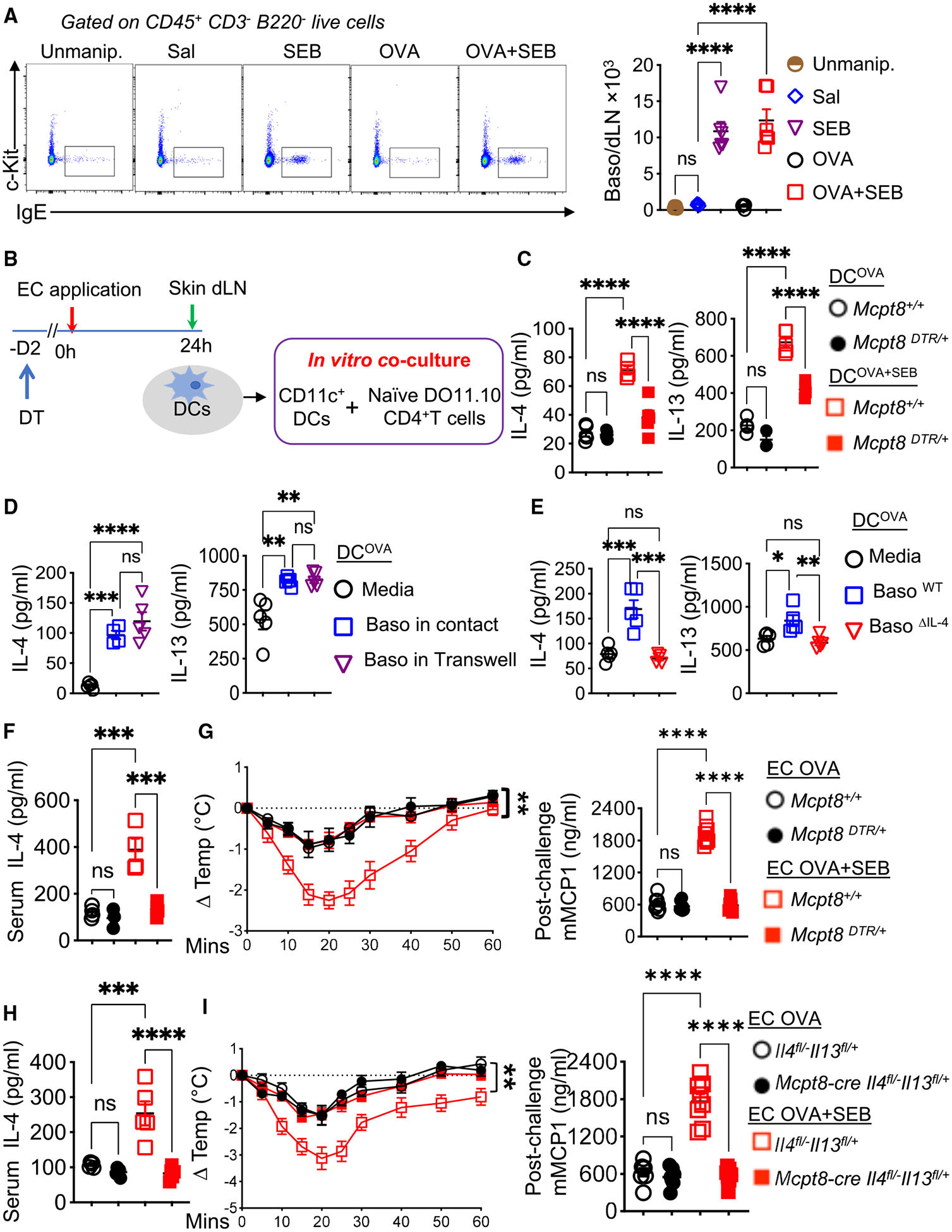
Basophils accumulate in dLNs of SEB-exposed skin, potentiate Th2 polarization by skin dLNe DCs, and enhance passive oral anaphylaxis in OVA + SEB-sensitized mice (A) Representative flow cytometry analysis and numbers of CD45^+^CD3^−^B220^−^c-kit^−^IgE^+^ basophils from skin dLNs of epicutaneously sensitized mice and Unmanip. controls, 4–6 mice/group. (B) Experimental protocol. (C) IL-4 and IL-13 secretion by naive CD4^+^ T cells co-cultured with DC^OVA^ or DC^OVA + SEB^ from DT-treated *Mcpt8*^*DTR/+*^ mice and *Mcpt8*^+/+^ controls. (D) IL-4 and IL-13 secretion by naive CD4^+^ T cells stimulated with DC^OVA^ and co-cultured with basophils from dLNs of SEB-exposed skin or separated from these basophils by a Transwell membrane, 4–5 mice/group. (E) IL-4 and IL-13 secretion by naive CD4^+^ T cells co-cultured with DC^OVA^ and Baso^ΔIL-4^ or Baso^WT^ controls, 4–5 mice/group. (F–I) Serum IL-4 concentrations (F and H), and post-challenge change in body temperature (G and I) (left), and serum mMCP-1 concentrations (G and I) (right) in DT-treated epicutaneously sensitized *Mcpt8*^*DTR/+*^ mice and *Mcpt8*^+/+^ controls (F and G) and in *Mcpt8-cre Il4*^*flox/−*^*Il13*^*flox/+*^ mice and *Il4*^*flox/−*^*Il13*^*flox/+*^ controls (H and I) subjected to passive oral anaphylaxis, 4–5 mice/group. A representative experiment out of 2 is shown for (A)–(F) and (H), and a pool of 2 experiments is shown for (G) and (I). Data are presented as mean ± SEM. *p < 0.05, **p < 0.01, ***p < 0.001, ****p < 0.0001 by one-way ANOVA with Tukey’s post hoc analysis in (A), (C)–(F), (G) (right), (H), and (F) (right) or repeated-measures one-way ANOVA (the Geisser-Greenhouse correction) in (G) and (I) (left); ns, not significant. See also Figure S6.

Basophils are a major source of IL-4 and play an important role in the Th2 cell response to cutaneous sensitization.^69–72^ Exposure of tape-stripped skin to OVA + SEB or SEB alone, but not OVA alone, or saline, elicited 24 h later a 8- to-10-fold increase in the numbers of CD45^+^CD3^−^B220^−^CD117 IgE^+^ basophils in the dLNs compared with dLNs of unmanipulated skin (Figure 6A). Basophil accumulation began as early as 6 h, peaked at 24 h, and waned by 48 h (Figure S6A). Basophil numbers in the spleen were comparable among all five groups (Figure S6B).

We next examined the effect of depleting basophils on Th2 polarization by DCs from skin dLNs. Diphtheria toxin (DT) injection 2 days before skin exposure to OVA + SEB (Figure 6B) effectively depleted basophils in skin dLNs of mice that express the DT receptor (DTR) under the control of the basophil selective *Mcpt8* promoter (*Mcpt8*^*DTR/+*^ mice) but had no significant effect in *Mcpt8*^+/+^ controls (Figure S6C). DT treatment significantly decreased the ability of DC^OVA + SEB^, but not DC^OVA^, from *Mcpt8*^*DTR/+*^ mice to drive IL-4 and IL-13 secretion by naive T cells (Figure 6C). DT treatment had no effect on Th2 cell polarization by DC^OVA + SEB^ or DC^OVA^ from *Mcpt8*^+/+^ controls (Figure 6C). These results indicate that basophils are critical for the enhanced Th2 cell polarization by DCs from dLNs of OVA + SEB-exposed skin.

### IL-4 from basophils potentiates Th2 cell polarization and passive oral anaphylaxis in mice epicutaneously sensitized to OVA + SEB

Basophils are a rich source in IL-4, which drives Th2 cell polarization. We investigated whether basophils sorted from dLNs of SEB-exposed skin potentiate Th2 cell polarization. Addition of these basophils to co-cultures of DC^OVA^ and naive CD4^+^ T cells resulted in a significant increase in IL-4 and IL-13, but not IL-17A and IFN-γ, secretion compared with co-cultures of DC^OVA^ and T cells alone (Figures 6D and S6D). IL-4 and IL-13 were not detectable when basophils were cultured alone, with DCs, or with naive CD4^+^ T cells. Basophils from dLNs of SEB-exposed skin enhanced Th2 cell polarization when separated from cultures of DCs and T cells by a Transwell membrane (Figure 6D), indicating that their ability to potentiate Th2 cell polarization was mediated by a soluble factor(s).

To investigate whether IL-4 derived from basophils mediates their enhancement of Th2 cell polarization, we sorted basophils from the dLNs of SEB-exposed skin from *Mcpt8-cre*^*Tg/0*^*Il4*^*flox/−*^*Il13*^*flox/+*^ mice, which selectively lack IL-4 in basophils (Baso^ΔIL-4^) and *Il4*^*flox/−*^*Il13*^*flox/+*^ controls (Baso^WT^). In contrast to Baso^WT^, Baso^ΔIL-4^ failed to potentiate Th2 polarization when added to co-cultures of DC^OVA^ and naive DO11.10 D4^+^ T cells (Figure 6E).

DT treatment on days −2 and 5 of epicutaneous sensitization abolished the increase in serum IL-4 concentrations and severity of passive oral anaphylaxis in *Mcpt8*^*DTR/+*^ mice, but not *Mcpt8*^+/+^ controls, sensitized to OVA + SEB (Figures 6F and 6G). By contrast, DT injection of OVA + SEB-sensitized *Mcpt8*^*DTR/+*^ mice 5 and 7 days after completing sensitization had no effect on the severity of passive oral anaphylaxis (Figures S6E and S6F), indicating that the role basophils play in exaggerating passive oral anaphylaxis in OVA + SEB mice is exerted during sensitization. Neither basophil depletion during nor after sensitization had a detectable effect on passive oral anaphylaxis in *Mcpt8*^*DTR/+*^ mice sensitized with OVA alone (Figures 6F, 6G, S6E, and S6F).

We next examined whether basophil-derived IL-4 is sufficient to drive the exaggerated passive oral anaphylaxis in mice epicutaneously sensitized to OVA + SEB. In contrast to *Il4*^*flox/−*^*Il13*^*flox/+*^ controls, *Mcpt8-cre*^*Tg/0*^ *Il4*^*flox/−*^*Il13*^*flox/+*^ mice failed to demonstrate increased serum IL-4 concentrations or exaggerated passive oral anaphylaxis following epicutaneous sensitization to OVA + SEB compared with epicutaneous sensitization to OVA alone (Figures 6H and 6I). Passive oral anaphylaxis and serum IL-4 concentrations were comparable in mice of the two strains sensitized to OVA alone (Figures 6H and 6I). Thus, basophil-derived IL-4 plays a critical role in the enhanced Th2 polarization, increased serum IL-4 concentrations, and exaggerated oral anaphylaxis in mice epicutaneously sensitized to OVA in the presence of SEB.

### Basophil influx in dLNs of skin exposed to SEB depends on CD40 expression by keratinocytes and keratinocyte-derived IL-33

**Figure 7.**
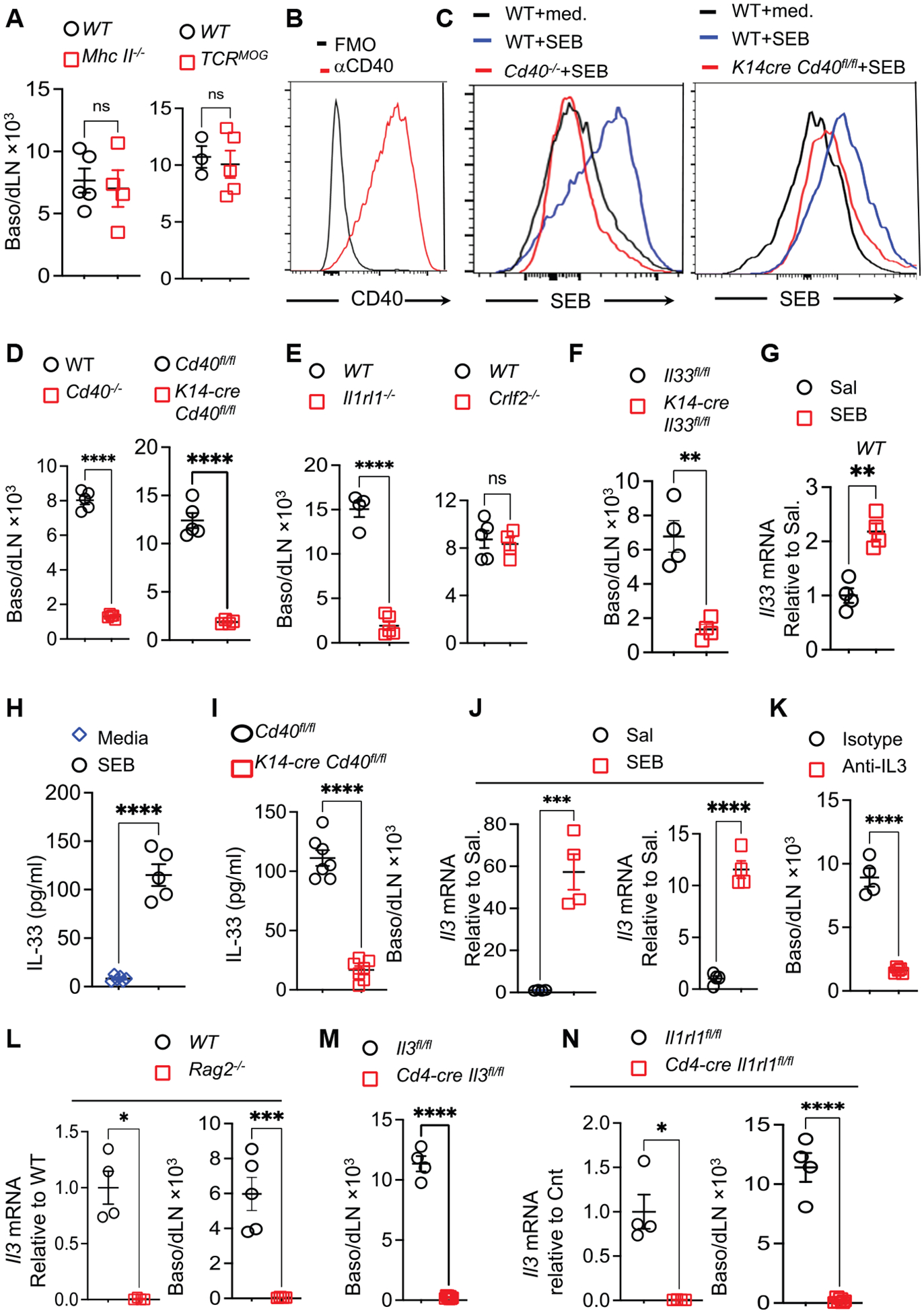
Basophil influx into dLNs of SEB-exposed skin depends on CD40 expression by keratinocytes, keratinocyte-derived IL-33, and T cell-derived IL-3 (A) Basophil influx in dLNs of SEB-exposed skin of *MhcII*^−/−^ mice, TCR^*MOG*^ transgenic mice, and WT controls, *n* = 3–5 mice/group. (B) Flow cytometry analysis of CD40 expression by CD45^−^EpCAM^+^ epidermal keratinocytes, 3–4 mice/group. (C) Flow cytometry analysis of SEB binding to keratinocytes from WT, *K14-cre Cd40*^*flox/flox*^ mice, and controls. Representative of 3–4 mice/group. (D–F) Basophil influx in dLNs of SEB-exposed skin of *Cd40*^−/−^ and *K14*-cre*Cd40*^*flox/flox*^ (D), *Il1rl1*^−/−^ and *Crlf2*^−/−^(E), and *K14-cre Il33*^*flox/flox*^ (F) mice and controls, 4–5 mice/group. (G) *Il33* mRNA expression in SEB-exposed tape-stripped skin of WT mice 4–5 mice/group. (H and I) IL-33 secretion by SEB-stimulated epidermal layers from WT (H) and *K14-cre Cd40*^*flox/flox*^ (I) mice, 5–7 mice/group. (J) *Il3* mRNA expression in total (left) and CD3^+^ T (right) cells from dLNs of SEB-exposed skin from WT mice, 3–4 mice/group. (K) Effect of anti-IL-3 on basophil influx in skin dLNs of SEB-exposed skin from WT mice, 4–5 mice/group. (L) *Il3* mRNA expression (left) and basophil influx (right) in dLNs of SEB-exposed skin from *Rag2*^−/−^ mice and controls, 4–5 mice/group. (M) Basophil influx in dLNs of SEB-exposed skin from *Cd4cre Il3*^*flox/flox*^ mice and controls, 4–5 mice/group. (N) *Il3* mRNA expression (left) and basophil influx (right) in dLNs of SEB-exposed skin from *Cd4cre Il1rl1*^*flox/flox*^ mice and controls, *n* = 4–5 mice/group. For all panels, a representative experiment out of 2 is shown. Data are presented as mean ± SEM. **p < 0.01, ***p < 0.001, ****p < 0.0001; ns, not significant by Student’s t test in (A), (B), (D)–(L) (right), and (M) and (N) (right) or Mann-Whitney t test in (L) (left) and (N) (left). See also Figure S7.

SEB binds to MHC class II and to the Vβ8 chain of the TCR.^73,74^ To examine whether the interaction of SEB with MHC class II and/or TCR Vβ8 is essential for basophil influx in dLNs, we used *MhcII*^−/−^ mice, which lack MHC class II, and *TCR*^*MOG*^ transgenic mice, whose T cells express exclusively *Tcrvb11*. Basophil influx in dLNs of SEB-exposed skin of MHC-deficient mice and *TCR*^*MOG*^ mice was comparable to WT controls (Figure 7A), indicating that SEB interactions with MHC class II and TCR Vβ8 are dispensable for this influx.

SEB induces chemokine production in CD40^+^ MHC class II^−^ human vaginal epithelial cells, and this is inhibited by silencing *CD40* expression using small interfering RNA (siRNA).^75^ Human keratinocytes express CD40.^76,77^ CD45 epithelial cell adhesion molecule (EpCAM)^+^ keratinocytes from WT mice, but not *Cd40*^−/−^ mice, expressed surface CD40 and *Cd40* mRNA (Figures 7B and S7A). Keratinocytes from WT mice, but not *Cd40*^−/−^ mice or *K14-creCd40*^*flox/flox*^ mice that lack CD40 expression in keratinocytes, bound biotinylated SEB (Figure 7C). Importantly, exposure of tape-stripped skin to SEB failed to induce basophil influx in the dLNs of *Cd40*^−/^ mice or *K14-creCd40*^*flox/flox*^ mice (Figure 7D). These results indicate that CD40-dependent SEB binding to keratinocytes is essential for basophil influx in dLNs of SEB-exposed skin.

IL-33 and thymic stromal lymphopoietin (TSLP) are epithelial cytokines released by keratinocytes following mechanical skin injury.^24^ Basophil influx in dLNs of SEB-exposed skin was drastically reduced in *Il1rl1*^−/−^ mice, which lack the IL-33R, as well as in *K14-creIl33*^*flox/flox*^ mice, but was not affected in *Crlf2*^−/−^ mice, which lack expression of *Crlf2*, which encodes the TSLP receptor (Figures 7E and 7F).

Application of SEB to tape-stripped skin of WT mice upregulated *Il33* mRNA compared with saline (Figure 7G). SEB also caused IL-33 to be released by epidermal layers from WT but not *K14-creCd40*^*flox/flox*^ mice (Figures 7H and 7I). p38 and extracellular signal-regulated kinase (ERK) drive *Il33* expression in keratinocytes.^78^ SEB-driven IL-33 release by epidermal layers of WT mice was abolished by pretreatment with inhibitors of p38, ERK, and c-Jun N-terminal kinase (JNK) but not nuclear factor κB (NF-κB) or Janus kinase (JAK), all of which are activated by CD40 ligation^79–82^ (Figure S7B). These results indicate that SEB-driven CD40-dependent release of IL-33 by keratinocytes is essential for basophil influx into the dLNs of SEB-exposed skin.

### IL-33-driven T cell release of IL-3 drives basophil influx into dLNs of SEB-exposed skin

IL-33 fails to cause the migration of purified basophils *in vitro*.^83^ By contrast, the cytokine IL-3, produced mostly by T cells,^84^ acts directly on basophils to cause their migration.^85,86^ *Il3* mRNA concentrations were significantly higher in dLNs, as well as in sorted CD3^+^ cells from dLNs of SEB-exposed skin compared with saline-exposed skin (Figure 7J). Importantly, basophil influx in the dLNs of SEB-exposed skin was markedly reduced by IL-3 blockade with anti-IL3 mAb, indicating an essential role for IL-3 (Figure 7K). *Il3* mRNA expression and basophil influx in the dLNs of SEB-exposed skin were virtually abolished in *Rag2*^−/−^ mice, which lack mature T and B cells but have normal numbers of basophils in the spleen (Figures 7L and S7C). Basophil accumulation in the dLNs of SEB-exposed skin was also virtually abolished in *Cd4creIl3*^*flox/flox*^ mice, which selectively lack IL-3 in T cells, compared with *Il3*^*flox/flox*^ controls (Figure 7M), but was intact in *Tcrd*^−/−^ mice (Figure S7D). *Il3* mRNA expression as well as basophil influx were also virtually abolished in the dLNs of SEB-exposed skin of *Cd4creIl1rl1*^*flox/flox*^ mice, which selectively lack expression of *Il1rl1*, encoding IL-33R, in T cells (Figure 7N). These results indicate that the basophil influx in dLNs of SEB-exposed skin is dependent on IL-33-driven release of IL-3 by T cells.

## DISCUSSION

We have unraveled mechanisms by which *S. aureus* skin colonization can aggravate food allergy in AD.

Food allergy was more frequent, and serum IL-4 concentrations were higher in AD patients with *S. aureus* skin colonization. Further, food allergy was associated with higher serum IL-4 concentrations in these patients. We investigated the relationship between *S. aureus* skin colonization, IL-4, and food allergy in a mouse model of AD elicited by epicutaneous sensitization with antigen in which oral antigen challenge results in IgE-mediated anaphylaxis.^47,48^ Sensitization to antigen in the presence of superantigen-producing *S. aureus*, or SEB, caused elevation of serum IL-4 concentrations and exaggerated oral anaphylaxis compared with sensitization with antigen alone. Of note, the densities of *S. aureus* and SEB we co-applied with antigen to the skin was comparable to those in skin lesions of AD patients colonized with SEB-positive *S. aureus*.^27^ Although OVA-specific IgE antibody concentrations were higher in mice sensitized with OVA in the presence of SEB compared with mice cutaneously sensitized with OVA alone, differences other than in IgE antibody contributed to the exaggerated oral anaphylaxis in these mice, as these mice demonstrated selectively exaggerated passive oral anaphylaxis.

Mice cutaneously sensitized with OVA + SEB had an increased antigen-specific Th2 cell response compared with mice sensitized with OVA alone. This was evidenced by greater secretion of IL-4 and IL-13 by OVA-stimulated splenocytes and higher serum IL-4 concentrations. Serum IL-13 concentrations did not increase. Sequestration of IL-13 by the decoy receptor interleukin-13 receptor subunit alpha-2 (IL-13Rα2), the expression of which is upregulated by IL-13, may explain this finding.^87–89^

Intestinal permeability was increased in OVA + SEB-sensitized mice compared with mice sensitized with OVA alone. IL-4, but not IL-13, blockade abolished both the increased intestinal permeability and exaggeration of oral anaphylaxis in OVA + SEB-sensitized mice. Although IL-13 plays a major role in mediating Th2 cell immunity, IL-4 plays a non-redundant role, and this is true in causing increased intestinal permeability.^18,90,91^ IL-4Rα blockade can attenuate food allergy in AD patients^92,93^ and improves the efficacy of peanut oral immunotherapy.^94^ Its effect on intestinal permeability has not been investigated.

T cells were the major source of IL-4 that mediated the heightened susceptibility to anaphylaxis in OVA + SEB-sensitized mice. We identified intestinal epithelial cells as the major target of IL-4 underlying the increase in intestinal permeability and susceptibility to oral anaphylaxis of OVA + SEB-sensitized mice, as both were abolished in mice with selective lack of IL-4Rα in intestinal epithelial cells. IL-4 decreased epithelial barrier resistance, increased intestinal permeability to histamine and prostaglandin E2,^18,91^ and increased transepithelial transport of HRP, OVA, and fluorescein isothiocyanate (FITC)-dextran conjugates in the T84 intestinal epithelial cell line.^53,95–97^ In contrast to our model, vascular endothelial cells are targets of IL-4 in mice in which administration of IL-4 immune complexes exaggerate anaphylaxis following intravascular anti-IgE challenge.^6^ This may be explained by differences in the route of challenge and possibly serum IL-4 concentrations.

DCs from dLNs of skin exposed to OVA + SEB selectively promoted Th2 cell polarization of naive OVA-specific T cells compared with DCs from dLNs of skin exposed to OVA alone. This was not due to increased antigen uptake or increased expression of MHC or co-stimulatory molecules CD80, CD86, or CD40. Rather, it was associated with increased IL-4R signaling with upregulation of several genes that promote Th2 cell polarization and downregulation of genes that inhibit Th2 cell polarization.

Skin exposure to SEB caused a robust increase of basophils in dLNs. IL-4 from these basophils potentiated DC-driven Th2 cell polarization and was essential for the increase in serum IL-4 concentrations and susceptibility to oral anaphylaxis in OVA + SEB-sensitized mice. This is consistent with the role basophils play in potentiating Th2 cell responses in allergy and helminth infections^98–100^ and of basophil-derived IL-4 in potentiating IgE sensitization and food allergy in mice cutaneously sensitized with OVA in the presence of the vitamin D3 analog MC903.^69^

SEB binds to MHC class II and TCR-Vβ8. Both were dispensable for the basophil influx in dLNs of SEB-exposed skin. Similar to human keratinocytes, mouse keratinocytes expressed CD40, and SEB binding to mouse keratinocytes was dependent on CD40. We uncovered a pathway by which CD40-dependent binding of SEB to keratinocytes caused the release of IL-33, which acted on T cells to drive expression of IL-3, which was essential for the basophil influx in dLNs of SEB-exposed skin. *S. aureus* skin infection augments IL-33 expression in the skin,^101^ IL-33 concentrations are elevated in lesional skin of patients with AD,^102^ superantigen-positive *S. aureus* causes more IL-33 release by human KCs compared with superantigen-negative *S. aureus*^103^ and IL-33 promotes food allergy.^104^ IL-33 is not a basophil attractant but enhances basophil migration to eotaxin.^83^ TSLP plays a critical role in the accumulation of basophils in MC903-treated skin independent of IL-3.^105^ TSLP played no detectable role in the basophil influx in dLNs of SEB-exposed skin.

A substantial proportion of food allergy patients fail to respond to currently available therapies; hence, there is an unmet therapeutic need in food allergy. We have generated foundational data that could lead to additional therapies for food allergy in patients with AD by targeting *S. aureus* skin colonization, CD40, IL-33, basophils, and IL-4 signaling.

### Limitations of the study

The CD11c^+^ cells used for Th2 polarization may include B220^+^ CD11c^+^ B cells in addition to DCs. The role of basophil-derived IL-4 in promoting the expression of IL-4/IL-13-driven genes by DCs in dLNs of skin exposed to OVA + SEB has not been directly established. The role of co-application of SEB with antigen in promoting the capacity of DCs from dLNs to drive Th2 cell polarization to antigens other than OVA needs to be established. The role of Paneth cells and goblet cells in the IL-4-dependent increase in intestinal permeability and exaggeration of oral anaphylaxis needs investigation. The molecular mechanism by which increased serum IL-4 enhances intestinal permeability remains to be elucidated.

## RESOURCE AVAILABILITY

### Lead contact

Further information and requests for resources and reagents should be directed to and will be fulfilled by the lead contact, Raif S. Geha (raif.geha@childrens.harvard.edu).

### Materials availability

This study did not generate new, unique reagents.

### Data and code availability

Any additional information required to reanalyze the data reported in this paper is available from the lead contact upon request.

## STAR★METHODS

### STUDY PARTICIPANT DETAILS AND EXPERIMENTAL MODEL

#### Study subjects

We studied 50 patients with AD and 8 healthy adult subjects. Thirty-six patients were recruited at Boston Children’s Hospital and fourteen at Denver National Jewish Hospital. The characteristics of the patients are shown in Table S1. Disease severity was assessed by SCORAD.^106^ Food allergy was diagnosed by a physician as noted in the patient’s record. Written informed consent from all subjects was obtained under a protocol approved by each center’s Institutional Review Boards. All samples were deidentified.

#### Mice

Wild type (WT) BALB/C and C57BL/6N mice were purchased from Charles River Laboratories. *Rag2*^−/−^ mice were purchased from Taconic. D011.10 TCR transgenic *MhcII*^−/−^, *TCR*^*MOG*^, *Cd40*^−/−^*, Tcrd*^−/−^ and *Pou2f3*^−/−^ mice on C57BL/6J background were purchased from Jackson Laboratory. *Igh7*^−/−^ mice were a gift from Dr. Hans Oettgen. *Mcpt5-cre, Rorα-cre, Il4ra*^*flox/flox*^*, Il1rl1*^−/−^, *Mcpt8*^*DTR/+*^ *Il1rl1*^*flox/flox*^,*Il33*^*flox/flox*^*, Il4/13*^*flox/flox*^ and *Crlf2*^−/−^ mice were obtained as previously described.^24,107,108^ *Mcpt5-cre* and *Rorα-cre* mice were crossed with *Il4/13*^*flox/flox*^ mice; all on BALB/C background. *Cd4-cre* mice on C57BL/6J were purchased from Taconic and crossed with WT mice on BALB/C mice for 8 generations, then crossed with BALB/c background *Il4/13*^*flox/flox*^ mice. *Cdh5-cre* mice were obtained from Dr. Simon Hogan.^109^ *Mcpt5-cre* and *Cdh5-cre* mice were crossed with *Il4ra*^*flox/flox*^ all on a BALB/C background. *Vil1-cre* mice on a C57BL/6J background were purchased from Jackson Laboratory and crossed with *Il4ra*^*flox/flox*^ mice on a BALB/C background for 9 generations. *Il3*^*flox/flox*^ mice on a BALB/C background were obtained from Dr Mei Li.^86^ *Cd40*^*flox/flox*^ mice on a C57BL/6J background were obtained from Dr. Kenneth M Murphy.^110^ *K14-cre* mice on a C57BL/6J background were purchased from the Jackson Laboratory and crossed with *Cd40*^*flox/flox*^ mice. *Mcpt8-cre* mice on a C57BL/6J background were purchased from The Jackson Laboratory and crossed with *Il4*^−/−^ mice on a BALB/C background from the Jackson Laboratory for 8 generations, then crossed with *Il4/13*^*flox/flox*^ mice on a BALB/C background. All mice were kept in a pathogen free environment. Procedures performed on mice were in accordance with the Animal Care and Use committee of Boston Children’s Hospital.

### METHOD DETAILS

#### Culture of human skin for *S. aureus*

Swabs from 2×2 cm areas of lesional skin were immersed in phosphate-buffered saline, then cultured on mannitol salt agar plates, and the plates examined after 48 hours of growth at 37 °C. *S. aureus* grew as yellow colonies or hyper-pink colonies on the mannitol salt agar plates. Colonies were verified as *S. aureus* by catalase and bound, clumping factor coagulase tests.

#### IgE concentrations in human serum

IgE concentrations in human sera were determined commercially using the ImmunoCAP technology by ThermoFisher.

#### *S. aureus* preparation and determination of skin load in mice

*S. aureus* COL and RN4220 strains were gifts from Dr. Patrick Schlievert. Single colonies were picked and inoculated in tryptic soy broth for overnight growth in a shaking incubator as previously described.^108^ The following morning 1:50 dilution of the bacterial suspension was inoculated in tryptic soy broth and cultured for another 2 hrs. Bacterial concentration was estimated by measuring absorbance at 600 nm. The bacteria were concentrated to 10^8^ CFU/50 μl of PBS and used for cutaneous application. CFUs were verified by overnight culturing of inoculum on Chrom-agar Plates.

To determine *S. aureus* load from the skin, an 8 mm^2^ punch biopsy was mechanically homogenized, and serial dilutions of the homogenate were plated on Chrom-agar plates. The plates were incubated overnight at 37 °C, and CFUs of *S. aureus* were enumerated based on the appearance of pink colonies.

#### Epicutaneous sensitization

Epicutaneous sensitization was performed as previously described.^47^ Briefly, 6–8 weeks old mice were anesthetized, and their back skin was shaved, and tape stripped with a film dressing (Tagaderm, 3M) 6 times at day 0, 3 times at day 2 and 2 times for other days. Epicutaneous sensitization consisted of applying a 1 cm^2^ gauze pad containing 200 μg OVA, 200 μg peanut or 10 μg SEB or OVA together with SEB or 10^8^ CFU of *S. aureus* or peanut together with SEB after each tape stripping on alternate days for 10 days.

#### Anaphylaxis

Mice were epicutaneously sensitized as described above. For active oral anaphylaxis, Mice were rested for 7–9 days after sensitization, then challenged with 150 mg OVA or peanut in 200 μL PBS. 24 h before challenge, mice were injected with an implantable temperature transponder (IPTT-300, BioMedic Data Systems). Core body temperatures were measured serially for 60 min after challenge using a DAS-6001 Smart Probe (Bio Medic Data Systems). After 60 min, blood was collected for serum analysis.

For passive oral and systemic anaphylaxis, epicutaneously sensitized mice were passively sensitized by *i.v*. injection of 10 μg IgE anti-trinitrophenyl (TNP) monoclonal antibody, a gift from Dr. Fred Finkelman.^111^ The following day, the mice were challenged by oral gavage of 2.5 mg TNP-BSA (T-5050, Biosearch) or *i.p*. injection of 8 μg TNP-BSA. Temperature monitoring and blood collection were done as detailed above.

For passive cutaneous anaphylaxis, epicutaneously sensitized mice were injected intradermally with 0.5 μg IgE anti-TNP monoclonal antibody in 30 μl of PBS into the right ear or PBS into the left ear. The following day mice were challenged with an *i.v*. injection of TNP-BSA (100 μg in 200 μl PBS) containing 1% Evans Blue dye. Vascular permeability was visualized 30 min later as blue pigmentation at the site of injection in the ears. To determine Evans Blue extravasation, the mice were euthanized and same-size pigmented areas around the ears injection sites were excised. The samples were immersed in formamide at 64°C for 12 hrs. Supernatant was collected and absorbance at 620 nm was used to determine Evans Blue extravasation.

#### Skin cell preparation

Skin pieces (1cm^2^) from epicutaneously sensitized mice were obtained and digested as previously described.^48,112^ Digested skin homogenates were filtered, washed resuspended in PBS and used for flow cytometry as previously described.^48,112^ For H&E staining, skin specimens were fixed in 4% PFA and embedded in paraffin. Sections (5 μm) of skin were stained with H&E stained by the Rodent Histopathology Core at Dana-Farber/Harvard Cancer Center.

#### Serum concentration of mMCP-1

Concentration of mMCP-1 in mouse serum were determined using an ELISA kit according to the manufacturer’s instructions (ThermoFisher).

#### Serum concentration of IgE anti-OVA antibodies

For detection of serum IgE anti-OVA antibodies, 96-well plates (ThermoFisher) were coated overnight at 4°C with rat anti-mouse IgE (clone R35–72, BD Biosciences) at 2 μg/mL overnight. The plate was blocked with 0.5% gelatin for 1 h. Serum samples were incubated for 2 h. Biotinylated-OVA at 12 μg/mL was used for detection. Bound OVA-biotin was detected with avidin-HRP (ThermoFisher) and color change after incubation with TMB substrate (ThermoFisher) was measured using a Biotek ELx808 plate reader.

#### Quantification of serum cytokines

Human serum IL-4 and IL-13 concentrations were determined by commercially available ELISA kits, according to the manufacturer’s instructions. Mouse serum cytokines were measured using the *in vivo* cytokine capture assay as previously described.^113^ Briefly, mice were *i.v*. injected with 10 μg of biotin- anti-IL-4 mAb (BVD6–24G2, eBioscience) or 10 μg of biotin-anti-IL-13 mAb (eBio1316H, eBioscience) and bled 16–24 hrs later. Serum IL-4 and IL-13 concentrations were determined by ELISA.

#### Cell culture and in vitro cytokine secretion

Single cell suspensions of splenocytes were cultured with 200 μg/ml of OVA for 4 days then supernatants were analyzed for cytokines by ELISA as previously described.^47,48^

#### Epidermal sheet preparation, culture and stimulation

Ears from mice were split into dorsal and ventral halves. To separate the epidermis and dermis, both halves were floated dermal side down on 4 mg/ml dispase (Gibco) in PBS for 30 mins at 37°C. Epidermal sheets were cultured in medium containing SEB (10μg/ml). The supernatants were harvested after 6 hrs and IL-33 was measured by an ELISA kit according to the manufacturer’s instructions (ThermoFisher).

For SEB binding assay with KCs, a single cell suspension of an epidermal sheet was incubated with biotinylated SEB (Toxin Technology) for 1 hr at 37°C, followed by flow staining with fluorochrome conjugated streptavidin.

#### Basophil depletion

*Mcpt8*^*DTR/+*^ mice and *Mcpt8*^+/+^ littermates on Balb/C background received an *i.p*. injection of 750 ng of diphtheria toxin (DT) per mouse.

#### *In vivo* antigen priming and analysis of mouse skin DCs

Antigen priming of skin DCs *in vivo* was performed as previously described.^107^ OVA (1 mg in 100 μL saline), SEB (10μg), OVA together with SEB, or saline were epicutaneously applied to the shaved and tape stripped back skin of mice. 24 hours later, DCs from axillary lymph nodes that had been enzymatically digested with 1mg/ml collagenase IV and DNase I were purified using CD11c beads from Miltenyi. Naïve (CD4^+^CD44^−^) CD4^+^ T cells were purified from spleens of DO11.10 mice after mechanical dissociation using the naïve CD4^+^T cells isolation kit from Miltenyi. 1×10^5^ DCs were co-cultured 1:1 with naïve CD4^+^CD62L^+^CD44^−^CD25^−^ DO11.10 T cells for 5 days without the addition of exogenous OVA.

For culturing basophils with DC and naïve DO11.10 CD4^+^ T cells, CD45^+^CD3^−^B220^−^c-Kit^−^IgE^+^ basophils were sorted from draining lymph nodes of SEB exposed skin using a Sony MA900 Cell Sorter. 10,000 basophils were added either together with the DC-T cells co-culture system or in the upper well of a Transwell system. Cell free supernatant were collected and mouse IL-4, IL-13, IL-17A, and IFNγ were detected by ELISA Ready SET Go kits (ThermoFisher).

For SEB binding assay with DCs, single cell suspensions of draining lymph nodes were incubated with biotinylated SEB (Toxin Technology) for 1 hr at 37°C, followed by flow staining with fluorochrome conjugated streptavidin.

#### RNA extraction and RT-PCR

RNA was extracted from homogenized draining lymph nodes and gut (1 cm) using the Qiagen Plus Mini RNA Extraction Kit (Qiagen). cDNA was synthesized using iScript^™^ cDNA Synthesis Kit (Bio-Rad Laboratories) and analyzed by TaqMan^®^ Universal Master Mix II and TaqMan Probes (Thermo Fisher) as detailed in the key resources table using a QuantStudio 5 (ThermoFisher) Real-Time PCR machine. Gene expression was normalized to *β2-microglobulin* using the 2^−ΔΔCt^ method. For each gene, the mean expression of the control was set to 1 for fold change calculations.

#### Transcriptomic analysis of dendritic cells from skin draining lymph nodes

RNA was extracted from CD11c^+^ DCs from skin draining lymph nodes as described above. cDNA was generated from 15 ng RNA using the SuperScript VILO cDNA synthesis kit (Invitrogen). Barcoded libraries were generated with the Ion AmpliSeq Transcriptome Mouse Gene Expression Panel (ThermoFisher) and sequenced with an Ion S5 system. Read alignment, de-multiplexing, quality control and normalization was performed with Torrent Suite. Differential gene expression was performed with Transcriptome Analysis Console software (ThermoFisher). Ingenuity Pathway Analysis was used for pathway analysis with the parameters specified in the text.

#### Jejunal cell preparation

Cells were isolated from the jejunum, duodenum and ileum as previously described.^24^ Briefly, jejunum, duodenum and ileum were harvested and flushed with PBS and 2% fetal calf serum (FCS) before being cut longitudinally and into 1 cm segments. The pieces were incubated in HBSS supplemented with 10 mM EDTA, 1.5 mM DTE, and 0.5% FCS shaking at 37°C for 40 min. Epithelial cells were collected from the supernatant and digested with Liberase DL (Roche) and DNase I (Sigma-Aldrich). Tissue pieces were then digested in HBSS with 20% FCS and 100 U/ml of collagenase VIII (Sigma-Aldrich) with shaking at 37°C for 30 min. Immune cells from the LP were further purified on a 40% Percoll gradient (GE Healthcare).

#### Flow cytometry

Cell suspensions were washed and incubated with TruStain FcX (anti-CD19/CD32, Biolegend) and eF506 viability dye (ThermoFisher) to exclude dead cells. Monoclonal antibodies to cell surface antigens were used for flow cytometry as detailed in the key resources table. All data were acquired on a BD LSRFortessa cell analyzer using FACSDiva software (BD Biosciences). Analyses were performed using FlowJo software (Tree Star, Inc.)

#### Intestinal permeability

Mice were gavaged with 400 μl of 5 mg/ml HRP (Sigma-Aldrich). Blood was obtained 20 min after gavage. Serum HRP content was measured by incubation with QuantaBlu Fluorogenic peroxidase substrate (ThermoFisher). Fluorescence intensity was measured by a BMG LABTECH FLUOstar Omega reader.

#### Treatment with antibodies to cytokines and CD4

Mice epicutaneously sensitized as described above were *i.p*. injected on day 16 and day 17 with 100 μg anti-IL-4 (11B11, BioXCell), anti-IL-13 (8H8, InvivoGen), anti-IFNγ (XMG1.2, BioXCell), anti-IL-17A (17F3, BioXCell) and combination of anti-IFNγ and anti-IL-17A or IgG1 isotype control. In another set, epicutaneously sensitized mice were *i.p*. injected day 16 and day 17 with 100 μg anti-CD4 (YTS 191, BioXCell) or IgG2b isotype control.

### QUANTIFICATION AND STATISTICAL ANALYSIS

All experiments were performed using randomly assigned mice without investigator blinding. Data were analyzed for normal distribution using the Shapiro-Wilk test. Anaphylaxis-related core body temperature measurements were analyzed using repeated measures one-way ANOVA (the Geisser-Greenhouse correction) with post hoc Tukey’s test for multiple comparisons or paired t-test. Depending on their distribution and number of groups, comparisons were analyzed for statistical significance using student’s t-test, Mann-Whitney t-test, one-way ANOVA with post hoc Tukey’s test, or Dunn’s test for multiple comparisons to determine the p-value using Prism software (GraphPad Software, Inc.). A p-value of <0.05 was considered significant.

## Supplementary Material

Supplementary Material

Supplemental information can be found online at https://doi.org/10.1016/j.immuni.2025.09.001.

## Figures and Tables

**Table:** KEY RESOURCES TABLE

REAGENT or RESOURCE	SOURCE	IDENTIFIER
Antibody		
IgE anti-trinitrophenyl	Gift from Dr. Fred Finkelman (Cincinnati Children’s Hospital)	N/A
Mouse IgE	BD Biosciences	Cat# 564207; RRID: AB-2738668
IgG1 isotype control	BioXCell	Cat# BE0088; RRID: AB_1107775
IgG2b isotype control	BioXCell	Cat# BE0090; RRID: AB_1107780
anti-mouse IL-4 Biotin	ThermoFisher	Cat# 13-7042-85; RRID: AB_466903
anti-mouse IL-13 Biotin	ThermoFisher	Cat# 13-7135-85; RRID: AB_763556
Anti-mIL-13-mIgG1 InvivoFit	InvivoGen	Cat# mil13-mab9-1
InVivoMAb anti-mouse IL-17A	BioXCell	Cat# BE0173; RRID: AB_10950102
InViboMAb anti-mouse IL-4	BioXCell	Cat# BE0045; RRID: AB_1107707
InViboMAb anti-mouse IFNγ	BioXCell	Cat# BE0055; RRID: AB_1107694
InViboMAb anti-mouse CD4	BioXCell	Cat# BE0119; RRID: AB_10950382
TruStain FcX (mouse CD19/CD32)	Biolegend	Cat# 101320; RRID: AB_1574975
mouse CD11b	BD Biosciences	Cat# 553309; RRID: AB_394773
mouse CD11c	Biolegend	Cat# 117303; RRID: AB_313772
mouse F4/80	Biolegend	Cat# 123105; RRID: AB_893499
mouse/human B220	Biolegend	Cat# 103204; RRID: AB_312989
mouse Gr-1	Biolegend	Cat# 108403; RRID: AB_313368
mouse NKp46	Biolegend	Cat# 137615; RRID: AB_11219387
mouse CD3	Biolegend	Cat# 100243; RRID: AB_2563946
mouse IgE	Biolegend	Cat# 406907; RRID: AB_493291
Streptavidin	Biolegend	Cat# 405229
CD45	Biolegend	Cat# 103116; RRID: AB_312981
CD117 (c-kit)	Biolegend	Cat# 105811; RRID: AB_313220
EpCAM	Biolegend	Cat# 118215; RRID: AB_1236477
CD45	Biolegend	Cat# 103112; RRID: AB_312981
CD4	Biolegend	Cat# 100548; RRID: AB_2563054
IL-4	Biolegend	Cat# 504106; RRID: AB_315320
SiglecF	Biolegend	Cat# 155506; RRID: AB_2750235
CD3	ThermoFisher	Cat# 46-0032-80; RRID: AB_1834428
Bacterial Strains		
S. *aureus COL*	Patrick Schlievert	N/A
*S. aureus RN4220*	Patrick Schlievert	N/A
Biological samples		
Human Plasma Samples	Samples were collected after informed consent in a research protocol approved by the Committee of Clinical Investigation at Boston Children’s Hospital, Hospita; and the National Jewish Hospital, Denver, CO	N/A
Chemicals		
Ovalbumin	Sigma-Aldrich	A5503
Peanut	nuts.com	Peanut Flour
Staphylococcal Enterotoxin B	Toxin Technology	BT202
Staphylococcal Enterotoxin B Biotinylated	Toxin Technology	BT202-B
Diphtheria toxin	Sigma-Aldrich	D0564
trinitrophenyl-bovine serum albumin (TNP-BSA)	Biosearch	T-5050
avidin-HRP	ThermoFisher	18-4200-89
TMB substrate	ThermoFisher	N301
Liberase DL	Roche	5989132001
Collagenase VIII	Sigma-Aldrich	C2139
Collagenase IV	Sigma-Aldrich	C4-28
Dispase	Gibco	17105-041
DNase I	Sigma-Aldrich	DN25
Percoll	GE Healthcare	17089101
viability dye	Thermofisher	65-0866
horseradish peroxidase	Sigma-Aldrich	P6782
QuantaBlu Fluorogenic peroxidase substrate	ThermoFisher	15169
Critical Commercial Assays		
Mouse IL-4 ELISA	ThermoFisher	88-7044
Mouse IL-13 ELISA	ThermoFisher	88-7137
Mouse IL-17A ELISA	ThermoFisher	88-7371
Mouse IFNg ELISA	ThermoFisher	88-7314
mouse MCPT1 ELISA	ThermoFisher	88-7503
mouse IL-33 ELISA	ThermoFisher	88-7333
Human IL-4 ELISA	ThermoFisher	88-7046
Human IL-13 ELISA	ThermoFisher	88-7439
Naïve CD4+ T cell Isolation kit, Mouse	Miltenyi Biotech	130-104-453
CD11c Microbeads, Mouse	Miltenyi Biotech	130-125-835
RNAeasy micro kit	Qiagen	74004
iScript cDNA synthesis kit	Biorad	1708890
TaqMan Universal Master Mix II, no UNG	ThermoFisher	4440043
SuperScript VILO cDNA Synthesis Kit	ThermoFisher	11754050
The Ion AmpliSeq Transcriptome Mouse Gene Expression Kit	ThermoFisher	A36553
Experimental Models		
*Mouse: MhcII* ^−/−^	Jackson Laboratory	RRID:IMSR_JAX:003584
*Mouse: TCR* ^*MOG*^	Jackson Laboratory	RRID:IMSR_JAX:006912
*Mouse: Cd40* ^−/−^	Jackson Laboratory	RRID:IMSR_JAX:002928
*Mouse: Igh7* ^−/−^	Gift from Dr. Hans Oettgen (Boston Children’s Hospital)	N/A
*Mouse: Balb/c Mcpt5-cre*	Talal Chatila, Boston Children’s Hospital	N/A
*Mouse: Il33* ^*flox/flox*^	Richard Lee	N/A
*Mouse: Il1rl1* ^*flox/flox*^	Richard Lee	N/A
*Mouse: CD4-cre* ^*Tg*^	Taconic	RRID:IMSR_TAC:4196
*Mouse: Il4* ^−/−^	Jackson Laboratory	RRID:IMSR_JAX:002496
*Mouse: Mcpt8*^*DTR/*^+	Hajime Karasuyama	N/A
*Mouse: Mcpt8-cre*	Jackson Laboratory	RRID:IMSR_JAX:017578
*Mouse: Vil1-cre*	Jackson Laboratory	RRID:IMSR_JAX:021504
*Mouse: DO11.10*	Jackson Laboratory	RRID:IMSR_JAX:003147
*Mouse: Pou2f3* ^−/−^	Jackson Laboratory	RRID:IMSR_JAX:037040
*Mouse: Tcrd* ^−/−^	Jackson Laboratory	RRID:IMSR_JAX:002120
*Mouse: Rag2* ^−/−^	Taconic	RRID:IMSR_TAC:RAG2
*Mouse: Cd40* ^*flox/flox*^	Kenneth Murphy	N/A
*Mouse: Il3* ^*flox/flox*^	Mei Li	N/A
*Mouse: Cdh5-cre*	Simon P Hogan	N/A
*Mouse: Balb/C Il1rl1* ^−/−^	Andrew N.J. Mckenzie	N/A
*Mouse: Il4ra* ^*flox/flox*^	Frank Brombacher	N/A
*Mouse: Balb/C Crlf2* ^−/−^	Steve F. Ziegler	N/A
*Mouse: K14-cre*	Jackson Laboratory	RRID:IMSR_JAX:018964
*Mouse: Il4-Il13* ^*flox/flox*^	Jackson Laboratory	RRID:IMSR_JAX:015859
*Mouse: Rora-cre* ^*Tg*^	Gift from Dr. Dennis O’Leary, California, USA	N/A
*Mouse: C57BL/6N*	Charles River	RRID:MGI:2159965
*Mouse: BALB/C*	Charles River	RRID:MGI:2161072
Software and Algorithms		
FlowJo	Tree Star, Inc	Version 10.8
Prism	GraphPad	Version 10
Oligonucleotides		
*B2m* Taqman Assays	Thermo Fisher	Mm00437762_m1
*Mcpt1* Taqman Assays	Thermo Fisher	Mm00656886_g1
*Mcpt2* Taqman Assays	Thermo Fisher	Mm00484932_m1
*Mcpt4* Taqman Assays	Thermo Fisher	Mm07306493_g1
*Il33* Taqman Assays	Thermo Fisher	Mm00505403_m1
*Il3* Taqman Assays	Thermo Fisher	Mm00439631_m1
*Il4* Taqman Assays	Thermo Fisher	Mm00445259_m1
*Cd40* Taqman Assays	Thermo Fisher	Mm00441891_m1

## Highlights

- SEB causes basophil influx in draining lymph nodes via a CD40 > IL-33 > T cell > IL-3 pathway
- Basophil-derived IL-4 promotes Th2 polarization by antigen-laden dendritic cells
- Increased IL-4 targets intestinal epithelial cells to enhance intestinal permeability
- Increased antigen absorption enhances mast activation, exaggerating anaphylaxis
