# Supplementary material for: S. aureus exposure during cutaneous antigen sensitization causes basophil- and interleukin-4-dependent exaggerated food anaphylaxis
Source: Immunity. Author manuscript; Available in PMC 2026 Jan 15. (PMC12805839; doi:10.1016/j.immuni.2025.09.001)
Supplement: Supplementary Material [file NIHMS2135431-supplement-Supplementary_Material.pdf]

Immunity, Volume *58*

Supplemental information

S. aureus exposure during cutaneous antigen sensitization causes basophil- and interleukin-4-dependent exaggerated food anaphylaxis

Mrinmoy Das, Mohammed Alasharee, Brian Woods, Saikat Mukherjee, Shira Kim, Megan Elkins, Jacqueline Ngo, Logan Magin, Maheshwor Timilshina, Juan Manuel Leyva-Castillo, Kenneth M. Murphy, Robert M. Anthony, Ana Flávia Santarine Laureano, George F. Murphy, Shannon McNamee, Frank Brombacher, Simon P. Hogan, Jerrold R. Turner, Shabnam Abtahi, Wanda Phipatanakul, Donald Y.M. Leung, Elena Goleva, Hans C. Oettgen, Mei Li, Janet Chou, Patrick M. Schlievert, Fred D. Finkelman, and Raif S. Geha

SUPPLEMENTARY FIGURES

Table S1. Patient characteristics

Pt. number	Sex	Age	SCORAD	*S. aureus* skin colonization	Food Allergy
1	Female	22	21.9	No	No
2	Female	27	21.9	No	No
3	Male	11	22.1	No	No
4	Female	32	22.8	No	No
5	Female	35	24.3	No	No
6	Female	33	31.6	No	No
7	Female	25	31.7	No	No
8	Female	15	32.7	No	No
9	Male	65	36.4	No	No
10	Male	23	46.9	No	No
11	Female	23	49.1	No	No
12	Female	29	26.9	No	Yes
13	Female	44	50.7	No	Yes
14	Female	12	51.7	No	Yes
15	Female	43	54.6	No	Yes
16	Female	26	24.9	Yes	No
17	Female	10	31.9	Yes	No
18	Male	4	20.8	Yes	No
19	Male	13	20.8	Yes	No
20	Male	17	22.4	Yes	No
21	Female	30	34.2	Yes	No
22	Female	10	36.6	Yes	No
23	Female	26	39.7	Yes	No
24	Male	14	40.2	Yes	No
25	Female	19	40.7	Yes	No
26	Female	13	55.8	Yes	No
27	Female	5	58.6	Yes	No
28	Male	11	63.5	Yes	No
29	Female	22	35.3	Yes	Yes
30	Female	10	27.9	Yes	Yes
31	Female	30	38.2	Yes	Yes
32	Female	26	17.8	Yes	Yes
33	Female	24	32.2	Yes	Yes
34	Female	6	39.8	Yes	Yes
35	Female	22	41.1	Yes	Yes
36	Female	26	43.8	Yes	Yes
37	Female	22	44.1	Yes	Yes
38	Female	17	45.8	Yes	Yes
39	Male	9	46.3	Yes	Yes
40	Female	23	47.8	Yes	Yes
41	Female	9	51.3	Yes	Yes
42	Female	8	55.4	Yes	Yes
43	Male	7	32.3	Yes	Yes

44	Male	5	55.8	Yes	Yes
45	Male	10	57.3	Yes	Yes
46	Female	33	25.2	Yes	Yes
47	Female	9	60.5	Yes	Yes
48	Male	11	65.9	Yes	Yes
49	Female	9	67.6	Yes	Yes
50	Female	22	47.3	Yes	Yes

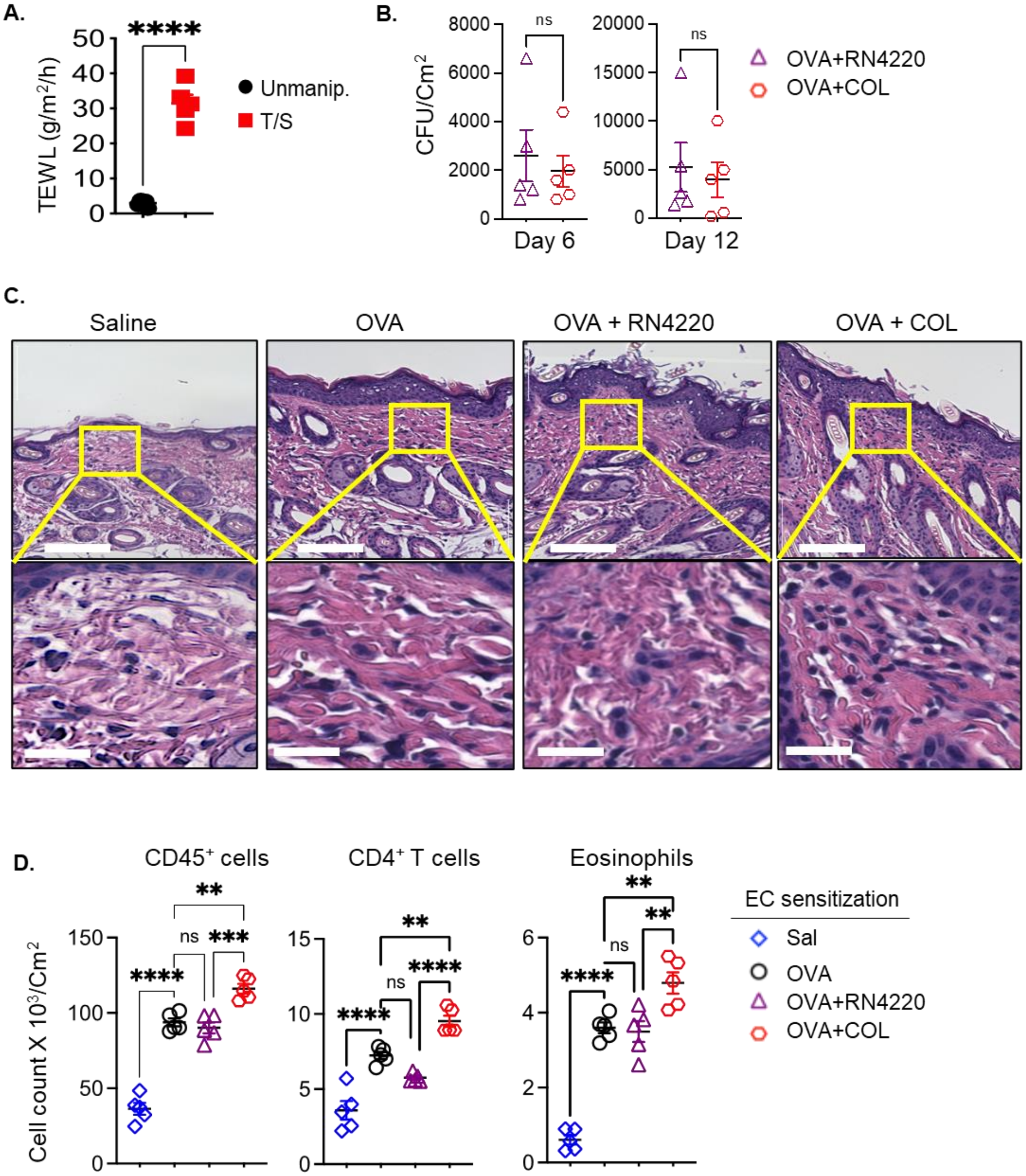

Figure S1. Effect of tape stripping on transepidermal water loss and *S. aureus* CFUs, histology and cell infiltration in tape stripped skin exposed to OVA and *S. aureus*. (Related to Figure 1) Transepidermal water loss in unmanipulated (Unmanip.) skin and immediately after tape stripping (T/S) for six times in WT BALB/C mice,4-5 mice/group. **B.** Representative photomicrographs at 40x magnification of H&E stained sections from tape stripped skin of WT mice sensitized with saline, OVA, OVA + *S. aureus* RN4220 strain, or OVA + *S. aureus* COL strain. Top: Scale bar 125 µm. Bottom: magnified field Scale bar 30 µm. **B.** Number of $CD45^+$ cells, $CD4^+$ cells and Eosinophils infiltrating 1 cm^2 of epicutaneously sensitized skin in WT BALB/C mice, 5 mice/group. Data are presented as mean ± SEM. * p <0.05; ** p <0.01; *** p

<0.001; **** p <0.0001 ns, not significant by two-tailed unpaired Student’s t-test in A and B and one-way ANOVA in B-G (right) with Tukey’s post hoc analysis in D.

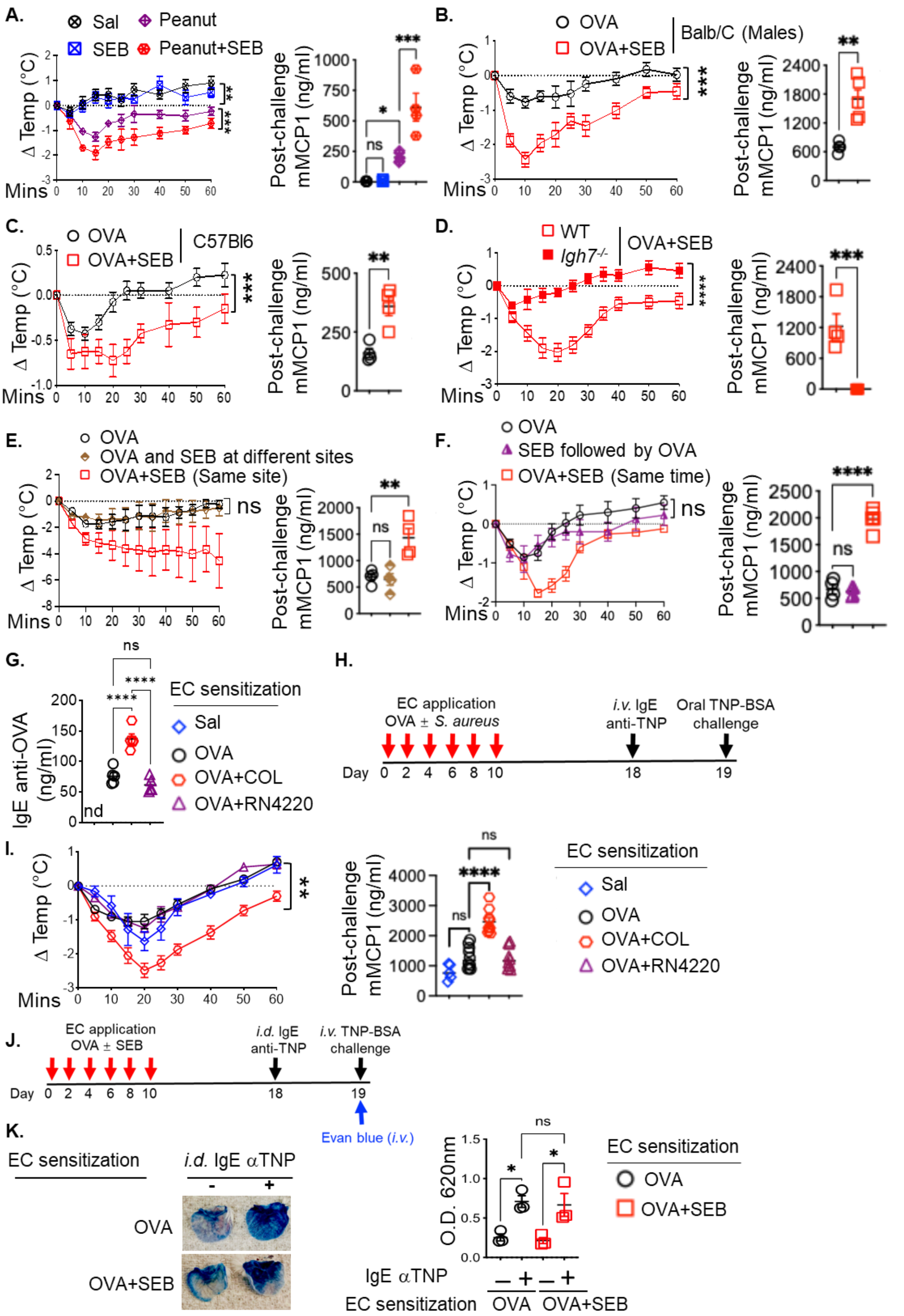

Figure S2. Epicutaneous sensitization to antigen and SEB exaggerates food anaphylaxis in a sex- and strain-independent, but IgE- and simultaneous co-exposure and time dependent manner, but and does not exacerbate passive systemic or cutaneous anaphylaxis. (Related to Figure 2). A-C. Change in body temperature 0-60 min post OVA challenge (left) and serum mMCP-1 concentration 60 min post-challenge (right) in BALB/C male mice (B), C57Bl/6J Female mice (C) and

Igh7$^{-/-}$ mice and WT BALB/C controls (D) epicutaneously sensitized with OVA alone or OVA-SEB, 4-5 mice/group. **D**. Change in body temperature 0-60 min post peanut antigen challenge (left) and serum mMCP-1 concentrations 60 min post-challenge (right) in mice epicutaneously sensitized as indicated, 4-5 mice/group. **E**. Change in body temperature 0-60 min post OVA challenge (left) and serum concentration of mMCP-1 60 min post challenge (right) in mice EC sensitized on back skin with OVA alone, or together with SEB applied to tape-stripped ear skin, 4-5 mice/group. **F**. Change in body temperature 0-60 min post OVA challenge (left) and serum mMCP-1 concentrations 60 min post challenge (right) in mice exposed to SEB on tape-stripped back skin then epicutaneously sensitized 14 days later with OVA at the same site, D 4-5 mice/group. **G.** Serum IgE anti-OVA concentrations in mice epicutaneously sensitized with OVA **i**n the presence of *S. aureus*. **H.** Experimental protocol**. I.** Change in body temperature post TNP-BSA challenge (left) and serum mMCP-1 concentrations 60 min post-challenge (right) in mice epicutaneously sensitized with OVA in the presence or absence of *S. aureus* then *i.v.* injected with IgE anti-TNP. **J**. Experimental protocol for epicutaneous sensitization and passive cutaneous anaphylaxis. **K.** Representative extravasation (left) and O.D. measurement of Evans blue (right) levels in ears of mice epicutaneously sensitized then intradermally (*i.d.*) injected with IgE anti-TNP followed by intravenous (*i.v.*) administration of TNP-BSA together with Evans blue, 3 mice/group. For A-G, I and K a representative experiment out of 2 is shown. Data are presented as mean ± SEM. $^*p<0.05$, $^{**}p<0.01$, $^{***}p<0.001$, $^{****}p<0.0001$ by paired t-test in in B-D (left), repeated-measures one-way ANOVA (the Geisser-Greenhouse correction) in A,E,F, I (left) or one-way ANOVA in A, E-G, I and K (right) with Tukey's post hoc analysis and Students t test in B-D (right). ns: not significant.

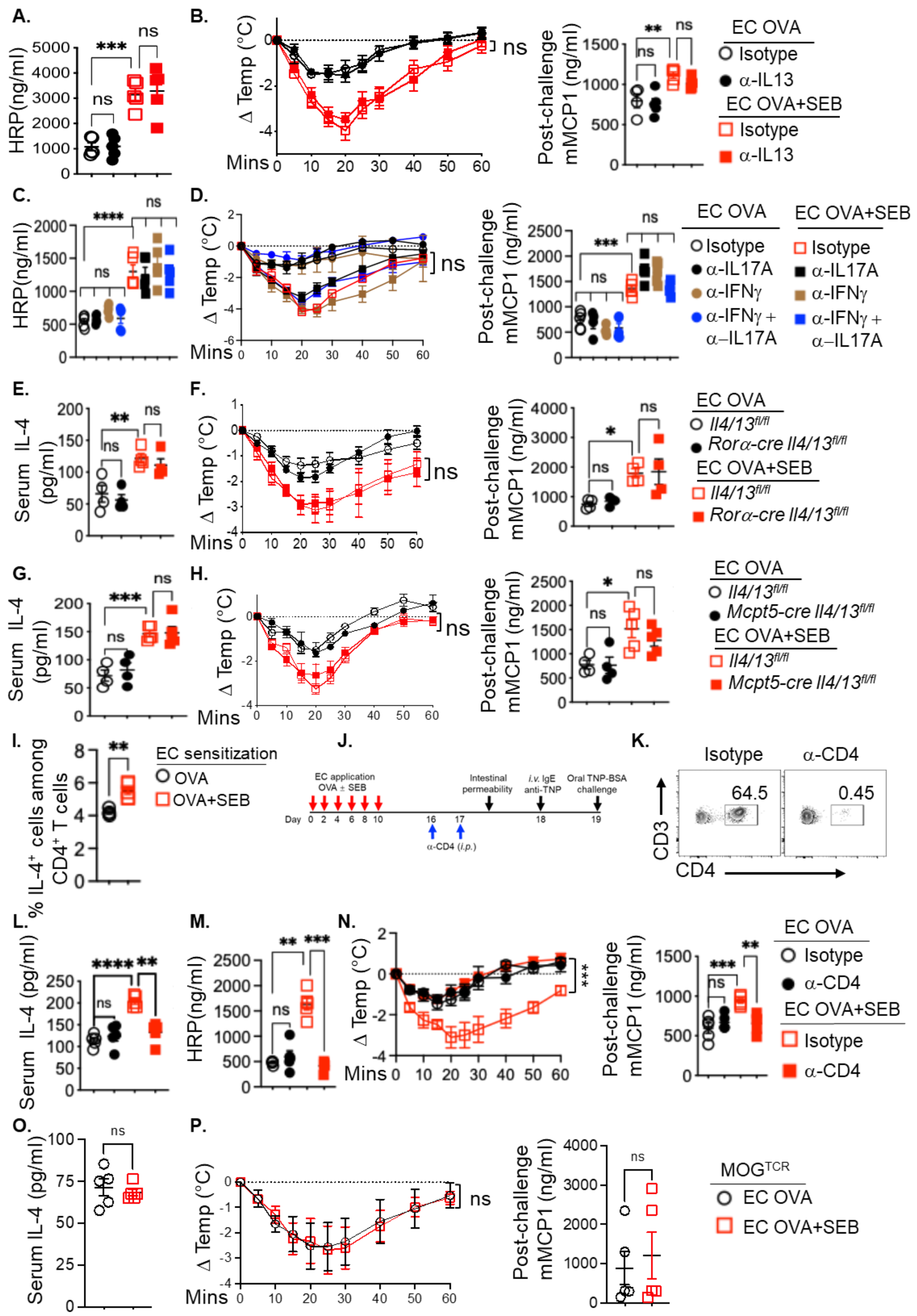

Figure S3. Intestinal permeability and passive oral anaphylaxis in OVA+SEB sensitized mice are independent of IL-13, IFNγ, anti-IL-17A and of IL-4 and IL-13 in ILC2s and MCs and are associated with antigen-dependent persistent activation of T heper-2 cells. (Related to Figure 3). A, B. Intestinal permeability assessed by measuring serum HRP concentrations after HRP gavage (A) and passive oral anaphylaxis assessed by change in body temperature (left) and serum concentrations of mMCP-1 (right) post-TNP-BSA oral challenge (B) in epicutaneously

sensitized mice treated with anti-IL-13 or IgG1 isotype control prior to passive sensitization and oral challenge, 4-5 mice/group. **C, D**. Intestinal permeability assessed by measuring serum HRP concentration after HRP gavage (C), and passive oral anaphylaxis assessed by change in body temperature (left) and serum concentration of mMCP-1 (right) post-TNP-BSA oral challenge (D) in epicutaneously sensitized mice treated with anti-IFNγ, anti-IL-17A, both or IgG1 isotype control prior to passive sensitization and oral challenge 3- 5 mice/group. **E-H**. Serum IL-4 concentration (E,G), and passive oral anaphylaxis with change in body temperature (F,H, left) and serum mMCP-1 concentrations (F,H, right) post TNP-BSA oral challenge in epicutaneously sensitized *Rorα-creIl4/13$^{flox/flox}$* mice (E, F) *Mcpt5-creIl4/13$^{flox/flox}$* mice (G,H) and *Il4/13$^{flox/flox}$* controls, 4-5mice/group. **I.** Percentages of splenic $CD4^{+}IL4^{+}$ cells on d19 in OVA+SEB sensitized, and OVA sensitized mice, 4mice/group. **J.** Experimental protocol for depleting $CD4^{+}$ cells in epicutaneously sensitized mice. **K.** Representative flow cytometry analysis of $CD4^{+}$ T cells gated on $CD45^{+}CD3^{+}$live cells from spleens of WT mice treated with anti-CD4 Ab or isotype control. **L-N.** Serum IL-4 concentration (L), intestinal permeability assessed by measuring serum HRP concentration after HRP gavage (M), and passive oral anaphylaxis assessed by change in body temperature (left) and serum concentration of mMCP-1 (right) post-TNP-BSA oral challenge (N) in epicutaneously sensitized mice treated with anti-CD4 mAb or IgG isotype control, 4-5 mice/group. **O, P.** Serum IL-4 concentrations (O) and change in body temperature (right) and serum concentration of mMCP-1 (left) post-TNP-BSA oral challenge (P) in epicutaneously sensitized MOG-TCR transgenic mice, 4-5 mice/group. For A-I, a representative experiment out of 2 is shown. Data are presented as mean±SEM. *p<0.05, **p<0.01, ***p<0.001, ****p<0.0001, ns: not significant by one-way ANOVA in A-D (right), E, F (right), G, H (right), L, M and N (right) with Tukey's post-hoc analysis or by repeated-measures one-way ANOVA (the Geisser-Greenhouse correction) in B, D,F,H and N (left) or Students t test in I, O, P (right) and by paired t-test in P (left).

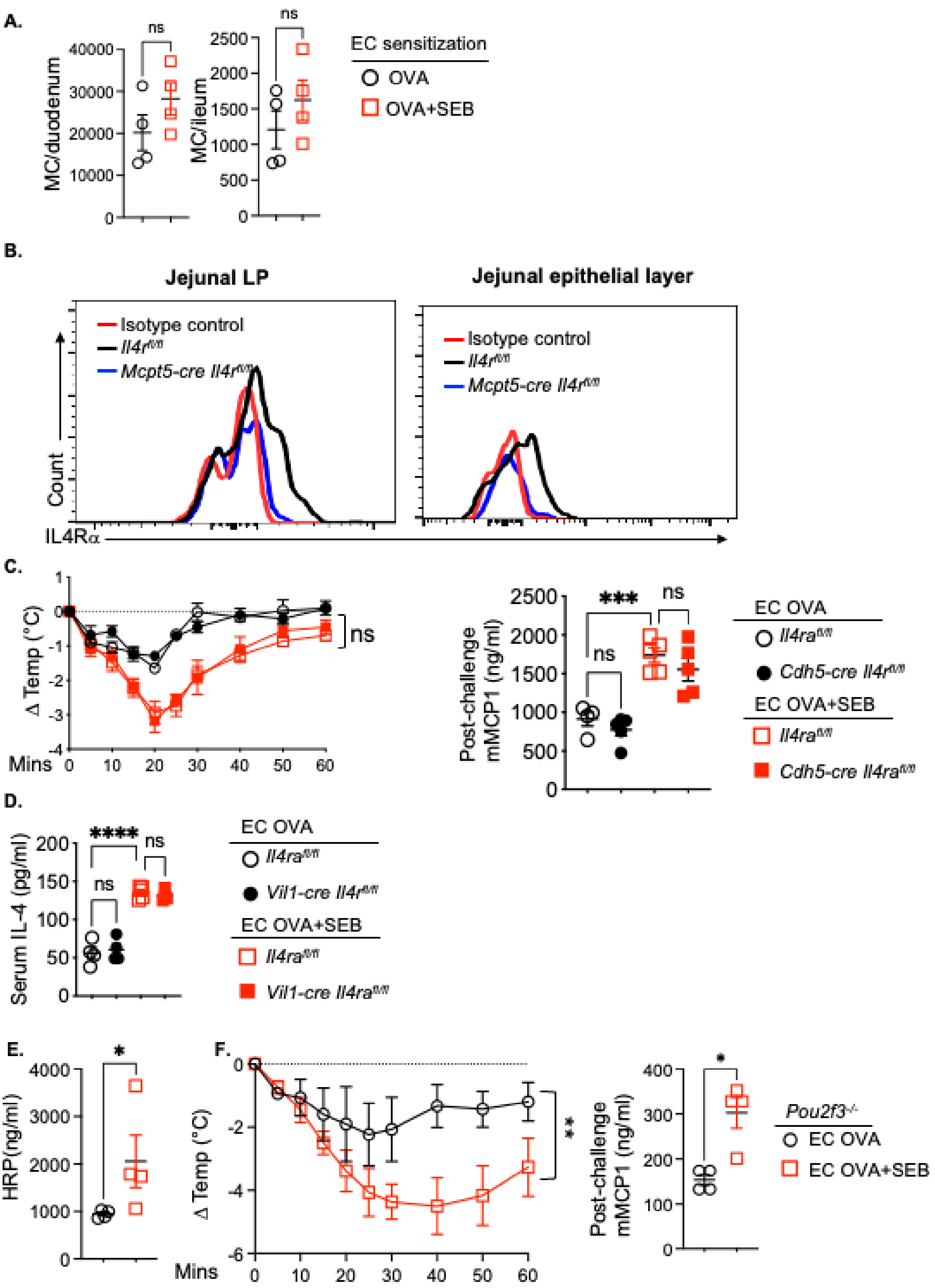

Figure S4. Intestinal MCs in epicutaneously sensitized mice, IL-4Rα expression by jejunal MCs, effect of deficiency of IL-4Rα in vascular endothelial cells and tuft cell deficiency on intestinal permeability and passive oral anaphylaxis. (Related to Figure 4). A. MC numbers in duodenum and ileum of OVA+SEB sensitized and OVA sensitized mice, 4 mice/group **B.** Representative flow cytometry analysis of IL-4Rα surface expression gating on MCs from the jejunal LP (left) and epithelial layer (right) of *Mcpt5-creIl4ra*$^{flox/flox}$ mice and *Il4ra*$^{flox/flox}$ controls. Isotype control staining used jejunal MCs from *Il4ra*$^{fl/fl}$ controls. **C.** Change in body temperature (left) and serum mMCP-1 concentrations (right) post challenge in epicutaneously

sensitized *Cdh5-creIl4ra*$^{flox/flox}$ mice and *Il4ra*$^{flox/flox}$ controls subjected to passive oral anaphylaxis, 4-5 mice/group. **D**. Serum IL-4 concentrations in epicutaneously sensitized *Vil1-creIl4ra*$^{flox/flox}$ mice and *Il4ra*$^{flox/flox}$ controls, n = 4-5 mice/group. **E,F**. Intestinal permeability assessed by measuring serum HRP concentration after HRP gavage (E), and change in body temperature (left) and serum concentration of mMCP-1 (right) post-TNP-BSA oral challenge (F) in epicutaneously sensitized *Pouf2/3*$^{-/-}$ mice, 4 mice/group. For B-D a representative experiment out of 2 is shown. Data are presented as mean±SEM. *p<0.05, ***p<0.001, ****p<0.0001, ns: not significant by Students t test in A, E and F (right) and by paired t-test in F (left), one-way ANOVA with Tukey's post-hoc analysis in C (right) and D, and repeated-measures one-way ANOVA (the Geisser-Greenhouse correction in C (left).

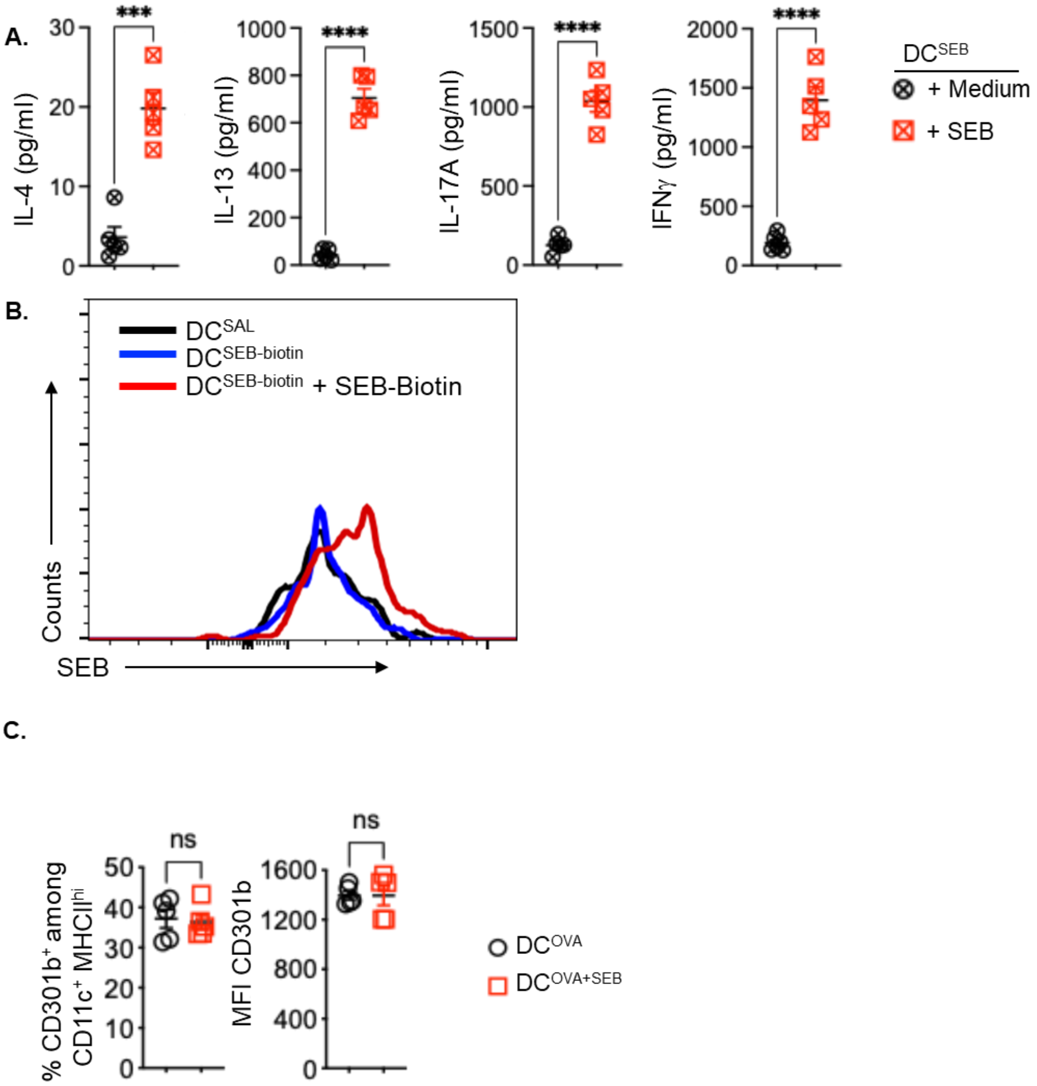

Figure S5. DCs from draining lymph nodes of tape stripped skin exposed to SEB cause cytokine secretion by DO11.10 cells only in the presence of exogenous SEB added to the culture. (Related to Figure 5). A. Cytokine secretion by naïve $CD4^{+}$T cells from DO11.10 mice co-cultured with $CD11c^{+}$ DCs isolated from draining lymph nodes of SEB exposed skin of mice in the absence (medium) or presence of SEB added to the culture, n=5 mice/group. **B.** Representative flow cytometry analysis of surface biotin on DCs from draining lymph nodes of tape stripped skin exposed to biotinylated SEB ($DC^{SEB\text{-}biotin}$) or saline (DC^{Sal}) 24 hrs earlier. As positive control, $DC^{SEB\text{-}biotin}$ were exposed for 1 hr *in vitro* to SEB-biotin washed and analyze. **C.** Percentage and MFI of $Cd301b^{+}$ cells among $CD11c^{+}MHCII^{high}$ $DC^{OVA+SEB}$ and DC^{OVA}, 5 mice/group. For A and C data are representative of two independent experiments. Data are presented as mean ± SEM. ***p<0.001, ****p<0.0001, ns: not significant by student t-test.

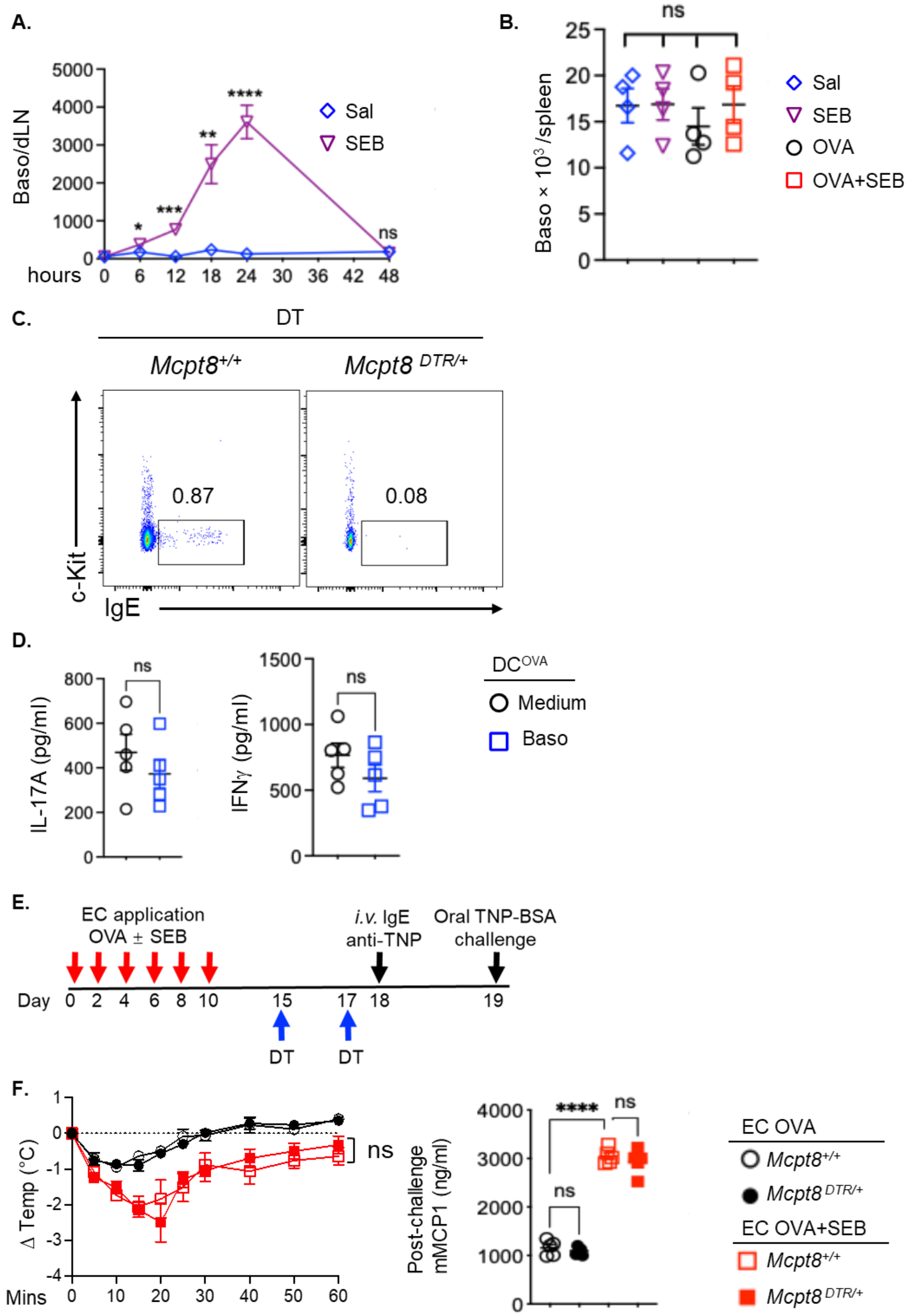

Figure S6. Kinetics of basophil accumulation in skin draining lymph nodes, lack of effect of added basophils from draining lymph nodes of SEB exposed skin on IL-17A and IFNγ production, and lack of effect of basophil depletion post epicutaneous sensitization with OVA+SEB on passive oral anaphylaxis. (Related to Figure 6). A. Kinetics of basophil influx in draining lymph nodes of SEB exposed skin of WT mice, n = 4-7 mice/group. **B.** Number of $CD45^{+}CD3^{-}$

$B220^-$c-$kit^-$$IgE^+$ basophils in the spleen of mice 24 hrs after exposure of tape-stripped back skin to OVA+SEB, OVA alone, SEB alone or saline, n = 4 mice/group. **C.** Representative flow cytometry analysis (left) and number (right) of basophils gated on $CD45^+CD3^-B220^-$ live cells from draining lymph nodes of SEB exposed tape-stripped skin $Mcpt8^{DTR/+}$ mice and $Mcpt8^{+/+}$ controls pre-treated with DT on Day -2, n = 4 mice/group. **D.** IL-17A (left) and IFNγ (right) secretion by naïve $CD4^+$ T cells from DO11.10 mice co-cultured *in-vitro* with DC^{OVA} and basophils from draining lymph nodes of tape-stripped skin exposed to SEB, n = 5 mice/group. **E**. Experimental protocol for DT treatment post epicutaneous sensitization of $Mcpt8^{DTR/+}$ mice and $Mcpt8^{+/+}$ controls. **F**. Change in body temperature (left) and serum mMCP-1 concentrations (right) post-challenge in epicutaneously sensitized $Mcpt8^{DTR/+}$ mice and $Mcpt8^{+/+}$ controls DT-treated post epicutaneous sensitization then subjected to passive oral anaphylaxis, n = 4-5 mice/group. For A, data are representative of two independent experiments; B, D and F, a representative experiment out of 2 is shown. Data are presented as mean ± SEM. **p<0.01, ***p<0.001, ****p<0.0001, ns: not significant by student t-test in A and D or by one-way ANOVA with Tukey's post hoc analysis in B and F (right) or by repeated-measures one-way ANOVA (the Geisser-Greenhouse correction) in F (left).

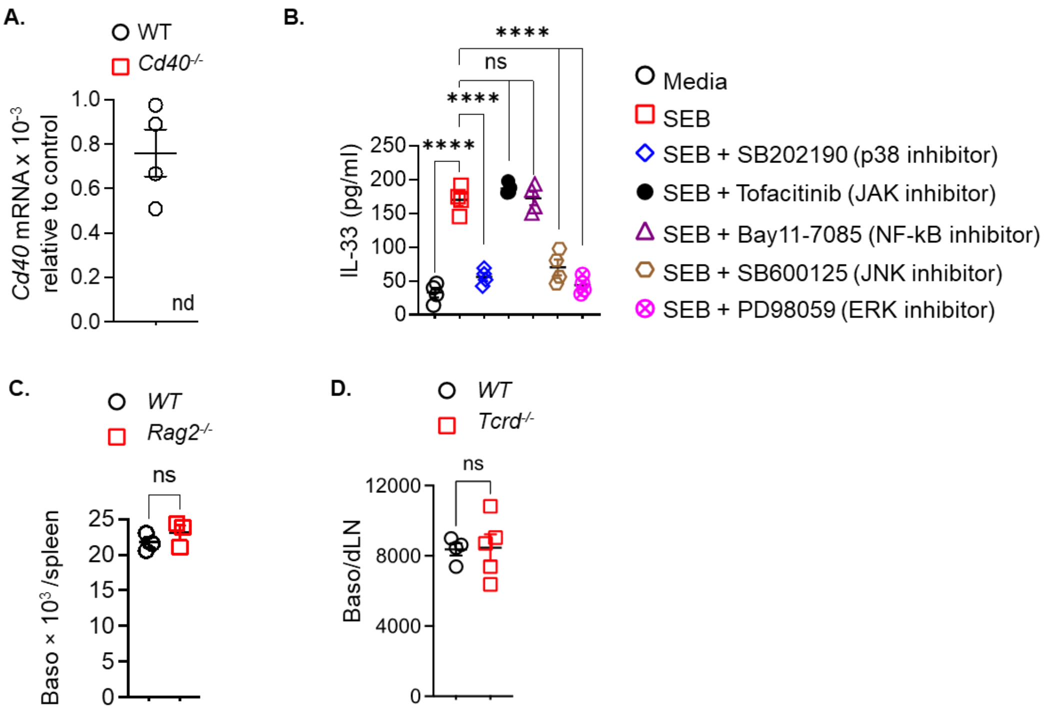

Figure S7. *Cd40* mRNA expression by keratinocytes, effect of inhibitors on SEB-driven IL-33 production by keratinocytes, spleen basophils in *Rag2$^{-/-}$* mice and basophil accumulation in draining lymph nodes of SEB-exposed skin from *Tcrd$^{-/-}$* mice. (Related to Figure 7) .A. qPCR analysis of *Cd40* mRNA expression by mouse CD45^{-}EpCAM^{+} keratinocytes sorted from the epidermal layer of *Cd40$^{-/-}$* and WT mice, nd: not detectable. 4/group. **B.** Effect of inhibitors on SEB-driven IL-33 production by epidermal layers from WT mice, 4/group. **C.** Basophil numbers in the spleens of *Rag2$^{-/-}$* mice and WT controls, 4 mic/group. **D.** Basophil numbers in draining lymph nodes of SEB-exposed skin of *Tcrd$^{-/-}$* mice and WT controls, 4-5/group Data are presented as mean±SEM. ****p<0.0001, ns: not significant by one-way ANOVA with Tukey's post hoc analysis in B and student t-test in C and D.
